# Intestinal Microbiome, Fecal Fermentation Profile, and Health Indices in HIV-Positive Men Versus Normal Controls Without HIV

**DOI:** 10.3390/nu18142328

**Published:** 2026-07-16

**Authors:** Mary C. Andreae, William A. Clark, John Sterrett, James Adkins, Jonathan P. Moorman, Brian M. Cartwright

**Affiliations:** 1 College of Health Sciences, East Tennessee State University, Johnson City, TN 37614, USA; clarkw@etsu.edu; 2 Quillen College of Medicine, East Tennessee State University, Johnson City, TN 37614, USA; cartwrib@etsu.edu; 3 Behavioral Neuroendocrinology Laboratory, Integrative Physiology, College of Arts and Sciences, University of Colorado, Boulder, CO 80309, USA; john.sterrett@colorado.edu; 4 Center of Excellence in Inflammation, Infectious Disease, and Immunity, East Tennessee State University, Johnson City, TN 37614, USA; adkinsjl1@etsu.edu (J.A.); moorman@etsu.edu (J.P.M.)

**Keywords:** human immunodeficiency virus, HIV, HAART, short chain fatty acids, fecal fermentation, fecal microbiome, dysbiosis, NAFLD, lipodystrophy, *Prevotella*, *Lachnospiraceae*

## Abstract

Background/Objectives: Many HIV-positive (HIV+) males receiving highly active antiretroviral therapy (HAART) experience metabolic complications, including non-alcoholic fatty liver disease (NAFLD); lipodystrophy; and intestinal dysbiosis, often characterized by a *Prevotella*-rich enterotype. Gut microbial fermentation produces short-chain fatty acids (SCFAs), which play important roles in host metabolism. This study investigated the relationships among HAART, anthropometrics, diet, intestinal permeability, gut microbiota composition, and lipodystrophy in HIV+ males. Methods: Forty males aged 23–60 years were enrolled, including 19 HIV+ participants recruited from the East Tennessee State University (ETSU) Health Infectious Diseases Specialty Clinic and 20 HIV-negative (HIV−) controls recruited through standard methods. Participants provided a stool sample for 16S rRNA gene sequencing, SCFA analysis by gas chromatography, and proximate analysis, and completed a food frequency questionnaire. Lipodystrophy-related measures included body mass index (BMI), hip-to-waist ratio (H:W), and liver health assessment using FibroScan. Blood samples were collected by venipuncture. Serum markers of intestinal permeability, including Claudin-21, flagellin, and intestinal fatty acid-binding protein (IFABP), were quantified by enzyme-linked immunosorbent assay (ELISA). Results: HIV+ males exhibited significantly higher H:W ratios (*p* = 0.001) and hepatic steatosis (*p* = 0.0047) than HIV− controls (Welsh’s *t*-test). Concentrations of isobutyrate (*p* = 0.0024), isovalerate (*p* = 0.0008), and valerate (*p* = 0.0329) were elevated in HIV+ participants, whereas butyrate (*p* = 0.0014) and total acetate/propionate/butyrate (APB) (*p* = 0.0046) were higher in HIV− males (Welsh’s *t*-test). HIV+ participants also showed greater abundances of *Prevotella* and *Lachnospiraceae* (Analysis of Compositions of Microbiomes; ANCOM). Retrospective analysis revealed that all HIV+ participants were men who have sex with men (MSM). Conclusions: HIV+ males demonstrated distinct gut microbiome profiles, altered SCFA production, and markers of disrupted lipid metabolism. These findings provide a foundation for future investigations of microbiome-metabolism interactions in HIV+ MSM.

## 1. Introduction

Human Immunodeficiency Virus (HIV) contraction continues to be a persistent health concern in the United States. In 2024, the Centers for Disease Control (CDC) reported that there are 1,158,701 people living with HIV in the United States, 77% of which are males aged 55–65 [1]. The CDC reported new diagnoses amounted to 38,793, 80% were men aged 25–34, of which 65% were men who have sex with men, and 6% contracted HIV via intravenous (IV) drug-related activities [1]. HIV was reported to account for 4296 deaths in 2024 alone; 77% of these deaths were men [1]. HIV is a virus transmitted through exposure to certain bodily fluids that targets CD4 T-cells, disrupting the body’s immune responses [1]. Without treatment, HIV may lead to Acquired Immune Deficiency Syndrome (AIDS), and possibly death. Current treatments for HIV include the use of Highly Active Anti-Retroviral Therapy, which can suppress viral replication and reduce viral loads to undetectable and non-transmittable levels [2,3]. HIV-positive individuals undergoing HAART exhibit metabolic abnormalities, specifically lipodystrophy and visceral adipose deposition [3,4,5,6,7,8,9]. As a result of these physiological changes, HIV-positive individuals are prone to developing metabolic syndrome and non-alcoholic liver disease (NAFLD), leading to liver and cardiovascular disease (CVD)—the two top leading causes of death in the HIV population undergoing HAART [7,8].

In addition to an altered metabolism, HIV-positive individuals may exhibit persistent malabsorption due to disruption of the gut barrier [8,9]. Additional populations, including HIV-positive individuals with NAFLD, have exhibited a leaky gut syndrome, described as increased intestinal permeability, bacterial translocation, increased cytokine production, and inflammation [8,10]. The gut microbiota has been found to be altered in individuals with leaky gut, as well as HIV, and may be contributing to metabolic abnormalities [8,9,10]. Intestinal fatty acid-binding protein (I-FABP)—a cytosolic protein found in enterocytes—as well as the presence of Lipopolysaccharide—a component of Gram-negative bacteria—and its’ corresponding immune marker, lipopolysaccharide-binding protein (LBP), are indicative of intestinal damage and translocation of bacteria, and are elevated in individuals with HIV infection [10,11,12]. Increased I-FABP and LBP have also been associated with an increase in liver fibrosis and NAFLD [12].

HIV replication is suppressed in most individuals undergoing HAART [13,14]. Nevertheless, persistent HIV infection activates immune responses, resulting in chronic inflammation in HIV-positive individuals [9,13,14]. Chronic inflammation is associated with metabolic disturbances, including insulin resistance and NAFLD [14]. Importantly, intestinal cells are a reservoir for latent HIV, and therefore may play a role in inflammation in the gut [11,13,14]. Interventions to decrease inflammation and improve malabsorption include prebiotics and probiotics, but have yielded mixed results in animal and human studies [15,16,17,18].

Overall, it is difficult to discern the roles HIV infection, HAART, and immune activation play concerning health outcomes in the population with HIV. One area of current interest is the composition of the intestinal microbiome and its relationship to HIV infection and comorbidities. It is known that a *Prevotella*-rich enterotype is associated with HIV and that changes in the microbiota would modify microbial metabolites, such as short-chain fatty acids, and the bidirectional communication between the gut and other cells [19,20,21,22]. It is well known that the composition and diversity of the human intestinal microbiome are complexly associated with human health and disease [22,23]. In healthy individuals, the intestine contains a variety of bacteria, bacteriophages, viruses, archaea, and fungi [24,25]. Alterations of the gut microbiome by environmental or host-cell factors can affect the bidirectional communication between microbial communities and host cells and play a meaningful role in energy regulation and substrate metabolism [21,22]. It is generally believed that better host health outcomes are associated with a balanced, diverse intestinal microbiota. Diet, body composition, genetics, lifestyle, sexual practices, and drug use influence microbial diversity and composition, as well as existing disease [23,25]. The composition of the microbiota directly influences microbial metabolites, including those involved in vitamin synthesis, choline and bile acid metabolism, and the fermentation of dietary constituents, which are utilized by host cells.

Products of fermentation of plant-based fibers by the gut bacteria produce short-chain fatty acids (SCFAs) of interest, C2-C7 in length, primarily in the large bowel [23]. SCFAs not only nourish enterocytes but also act as ligands through interactions with cognate G-protein-coupled receptors (GPCRs) in tissues and peroxisome proliferator-activated receptor (PPAR-γ) [23,26], initiating downstream effects on cell proliferation, differentiation, and gene expression [23]. Thus, the bacterial profile of fermentation products not only affects energy metabolism but also intestinal homeostasis, including intestinal permeability and immune response regulation.

Where straight SCFAs primarily induce a homeostatic state in tissues, branched SCFAs (BCFAs) may shift the balance. BCFAs result from protein fermentation in the gut, particularly the branched-chain amino acids (BCAAs) leucine, isoleucine, and valine. Increased BCAAs in the colon can alter the pH (increase) and microbial metabolism, supporting a more pro-inflammatory microbiome [27]. Dysbiotic enterotypes are correlated with a sustained pro-inflammatory state [28,29,30,31]. Inflammatory mechanisms can promote metabolic and immune abnormalities, including lipodystrophy, NAFLD, insulin resistance, and chronic and autoimmune diseases [29,32]. Related conditions include inflammatory bowel diseases (IBDs), rheumatoid arthritis (RA), HIV infection, metabolic syndrome, cardiovascular disease, and diabetes [28,29,32].

It is known that there is a wide range of intestinal microbiomes considered healthy. Yet, it is also well established that certain microbial fingerprints, or enterotypes, are associated with certain diseases (Table 1). Since communication between the intestinal microbiome and the body’s multiple systems is multidirectional and complex, it is unknown whether dysfunction originates in the gut or from host metabolic abnormalities. Regardless, there is an undeniable relationship between the microbial world and the human body.

In a healthy gut, commensal bacteria produce the predominant SCFAs—acetate, propionate, and butyrate—at a molar rate of 60:20:20, respectively [20]. Approximately 500–600 mmols per day are produced depending on fiber content in the diet, microbiota composition, and the gut’s transit time [34]. BCFAs, primarily isobutyrate, isovalerate, and valerate, are produced by fermentation of branched-chain amino acids at lower molar concentrations [35,36]. Butyrate is most beneficial to colonocytes and hepatocytes, providing 60–70% of their energy while acting as an anti-inflammatory mediator [23,37]. Most SCFAs not metabolized by the enterocytes are metabolized by hepatocytes, with the exception of acetate [20,38].

The bacterial microbiota can be segmented into four major phyla: *Firmicutes*, *Bacteroides*, *Proteobacteria*, and *Actinobacteria* [39]. Firmicutes and *Bacteroides* are predominant at approximately 98% of the total microbiota [39]; where *Bacteroides* primarily produce acetate and propionate, Firmicutes produce butyrate [39]. Within the phylum Firmicutes, specific clostridial clusters, the primary butyrate producers, account for 10–40% of the microbiota in a healthy gut [39,40]. BCFAs, such as isobutyrate, isovalerate, and valerate, are produced by the fermentation of BCAAs, primarily by the genera *Bacteroides* and *Clostridium* [37].

SCFAs and BCFAs interact with GPCRs; GPCR43, and GPCR41 in the apical interface of the intestinal epithelial cells and in immune cells in the lamina propria and mucosal lymphoid tissue [26]. GCPR109A in the lamina propria, is also expressed in adipocytes and immune cells [26,41]. GPCR activation via SCFA binding occurs in multiple cell types, including, but not limited to, intestinal cells, enteric neurons, endocrine cells, liver cells, adipose cells, and immune cells [26]. It is evident that there is a complicated metabolic interconnectedness between the intestinal microbiome, fecal fermentation products, and the underlying inflammation that may also be associated with latent HIV infection. Changes in the microbiome, the corresponding shifts in fermentation products, and modifications in adiposity in the HIV-positive population have yet to be investigated.

This study aimed to further elucidate the relationship between HIV infection; serum markers of gut permeability, intestinal microbiome, and fecal fermentation profiles; and measures of lipodystrophy in males with HIV. Our long-term goal is to help elucidate the relationship between these factors and seek out dietary interventions that will improve health outcomes in the HIV-positive population. Our central hypothesis is that HIV is positively correlated with changes in the microbiome, resulting in altered fecal fermentation profiles and visceral adiposity.

## 2. Materials and Methods

### 2.1. Recruitment and Initial Data Collection

Forty subjects were recruited for this study, 19 males with HIV and 21 males without HIV, between the ages of 18 to 60. HIV-positive males were recruited through the ETSU Health Infectious Disease Clinic in Johnson City, Tennessee, during routine patient visits. HIV− controls were recruited through standard procedures, including fliers and network-based outreach. At the time of recruitment, subjects signed informed consent (Supplementary File S1) and received a stool collection kit and a Block food frequency questionnaire (FFQ) (Supplementary File S2) [42,43]. Participants completed the 2014 Full-Length Food Frequency Questionnaire and Physical Activity Screener (127 food item list) to assess their caloric intake and typical nutrition component of interest (carbohydrate, protein, fat, fiber) intake (Nutrition Quest, Berkeley, CA, USA) [42,43,44]. Once completed, food frequency questionnaires were sent to Nutrition Quest for analysis. After analysis by Nutrition Quest, the results were compared using Welch’s *t*-tests. Anthropometric measures including height, weight, and hip and waist circumference [45], FibroScan (Westborough, MA, USA) (Acoustic Radiation Force Impulse; ARFI) ultrasound technique [46] (elastography)—which was performed by a trained technician—and data were collected, along with two serum separator tubes of blood collected via venipuncture Serum samples were centrifuged at room temperature for 10 min at 3000× *g*, decanted using a Pasteur pipette and transferred to a 3 mL amber glass vial and stored at −80 C until analysis. Subjects returned at the scheduled time to deliver their stool samples and complete the FFQ. Males without HIV were recruited via a convenience sampling method. All participants were given a $20.00 gift card for participation in the study, which was distributed at the initial visit.

### 2.2. Anthropometric Measures

Hip and waist circumferences were measured using the World Health Organization (WHO) protocol at the time of recruitment [45]. Height and weight were measured with a stadiometer for BMI calculation. FFQs were distributed at recruitment and collected when stool samples were returned to the clinic.

### 2.3. Liver Scans

Liver scans were measured via the acoustic variation force impulse (ARFI) ultrasound technique for hepatic fibrosis and hepatic steatosis. Fibrosis is measured in Liver Stiffness Measurement (LSM) measured in kilopascals (kPa), with staging for NAFLD being F0–F4; and hepatic steatosis is expressed as a Controlled Attenuation Parameter (CAP) in decibels per meter (dB/m), graded S1–S3 relative to the portion of liver infiltrated by fat [46,47].

### 2.4. Food Frequency Questionnaires

All 40 subjects completed food frequency questionnaires to address dietary variability. Validated Block 2014.1 1-month FFQ [42,43].

### 2.5. Stool Sample Preparation and Freeze-Drying

A 600 mL LABCONCO freeze dry flask were tared for each stool sample. Fecal samples were placed in the freeze dry flask. An initial wet weight was obtained for moisture loss. Freeze-drying LABCONCO FreeZone 2.5 freeze dryer (Kansas City, MO, USA) using stainless steel adapters was used. The samples were freeze-dried for at least 24 h on 0.077 mBar at −50 °C. Post-drying, the flask was weighed to determine the weight of water lost. Samples were ground and stored at −80 °C until analysis. For proximate stool macronutrient analyses all samples were analyzed in duplicates. The percent dry weight (DW) was determined with flask weight before and after using the formula:

(1) $$DW=1+\frac{\mathrm{Wet}\mathrm{weight}\mathrm{of}\mathrm{feces}}{DW}\times 100$$

### 2.6. Bomb Calorimetry

All analyses were performed using oxygen bomb calorimetry, Paar 6200 calorimeter, Paar instrument company (Moline, IL, USA) [48,49]. The combustion capsule was tared and 2.2 g of ground freeze-dried feces was placed in the capsule. The combustion capsule was suspended in the bomb vessel with the ignition thread completing the circuit between the bomb vessel and the dried fecal sample. The bomb vessel was then filled with oxygen. Two liters of deionized distilled water (DDW) was placed into the bucket, with ignition wires attached. Calories were calculated per gram of feces [49]. The bomb vessel was removed post-combustion from the bucket, and the bomb was rinsed with DI water which was collected and titrated back to neutral pH with sodium carbonate solution (0.0709 Nitrogen solution) until color change was sustained, to measure nitric acid formed during combustion [48]. The formula used to determine total digestible calories:

(2) Totaldigestiblecalories=total calories−mL of NaCO3

### 2.7. Proximate Analyses

Samples were subjected to proximate analysis using standardized methods for moisture (AOAC 930.15/925.10), ash (942.05), protein via Kjeldahl (984.13/920.87), and fat via Soxhlet using petroleum ether (920.39) [50]. Carbohydrate content was determined by difference:

(100 − % moisture − % ash − % protein − % fat)(3)

### 2.8. Fiber

Total dietary fiber (TDF), soluble dietary fiber (SDF), and insoluble dietary fiber (IDF) is assessed on freeze-dried, ground stool samples using the automated ANKOM Dietary Fiber Analyzer method AOAC 991.43 (Macedon, NY, USA) [51,52]. Reagents included 78% Ethanol by volume, alpha-amylase (5 mL/25 mL DDW), protease (5 mL/25 mL DDW), amyloglucosidase (5 mL/25 mL DDW), MES-TRIS buffer, and 0.561 N HCl. The MES-TRIS solution is prepared by dissolving 9.76 g of 2-(*N*-Morpholino) ethanosulfonic acid (MES) and 6.1 g of Tris(hydroxymethyl)aminomethane (TRIS) in 850 mL of DDW and adjusting the pH to 8.2 using 6 N NaOH and diluted to 1 L with DDW. ANKOM IDF and SDF filter bags are labeled, weighed, and recorded in a Dietary Fiber Data Spreadsheet (DFDS). One gram of diatomaceous earth is weighed into 6 boats for SDF analysis bags and 0.5 g of the freeze-dried ground stool sample was weighed in duplicate into 6 boats for the IDF analysis bags. The fiber analysis instrument performs an automated sample digestion process that is completed in approximately 3 h. After the amylase and protease phases, the pH of the samples is checked and adjusted to 4.0–4.7, as needed, using 0.561 N HCl. After the automated process is complete, IDF and SDF bags are rinsed with Acetone. After drying, the bags are sealed at a heat setting of 3 using the ANKOM Heat Sealer. Samples are then placed on a drying rack and in a Fisher Scientific Isotemp oven (Pittsburg, PA, USA) at 100 °C for 90 min. Samples are removed from the oven and immediately placed in a desiccator to cool. Bags are re-weighed after cooling. A protein correction is performed using kjeldhal digestion and distillation of the bags, as described in fecal % crude protein methods. An ash correction is also performed to account for mineral content of the samples. All ashing and protein values were recorded on the DFDS [51]. Calculations are as follows:

(4) $$\%IDF\;or\;\%\;SDF=\;\frac{\left[Total\;residue-\left(protein\;residue+bag\right)-(ash\;residue+bag\right)}{\left(Sample\;weight\;grams\;\times \;100\right)}$$

(5) %TDF=%IDF+%SDF

### 2.9. Short-Chain Fatty Acids

SCFA extractions are performed using a modification of a procedure developed by Schwiertz et al. [53]. One mL of the SCFA extraction solution, containing Oxalic acid (0.1 mol/L) and Sodium Azide (40 mmol/L), are added to 80 mg of a freeze-dried stool sample in a 16 mm × 100 mm disposable culture tube. The samples are vortexed for 30 s and then placed on a horizontal shaker for 1 h, then centrifuged at 4000 rpm for 20 min. After centrifuging, the supernatant is removed and placed in a 1.5 mL polypropylene micro-centrifuge tube. The solution is re-centrifuged at 12,000 rpm for 15 min. Again, the supernatant is removed and placed in a new 1.5 mL micro-centrifuge tube. The solution is re-centrifuged at 12,000 rpm for an additional 15 min. Finally, the supernatant is removed and placed in a 2 mL GC vial containing an insert and analyzed using a Shimadzu GC2010 gas chromatograph (Shimadzu Corporation, Kyoto, Japan) with SigmaAldrich ZB-Wax Plus capillary column (Phenomenex, Torrance, CA, USA). Samples are run using a method adapted from K. Schäfer [54]. The method includes injecting 1 mL of solution with an SPL1 temperature of 250 °C. The initial column temperature is 50 °C, held for 2 min, which rises at a rate of 15 °C/min until reaching 140 °C with a hold of 5 min, followed by a rise at rate of 10 °C/min until reaching 160 °C with a hold of 3 min, and a rise of 10 °C/min until reaching 175 °C with a hold of 3 min. The flame ionization detector temperature was 180 °C, and the end time of the run was 24 min. Samples were run in duplicate, and values for each participant were averaged.

### 2.10. Gut Permeability Measures

Tight junction markers (Claudin-21, FABP-I) [55,56,57], and markers of bacterial translocation (Flagellin) [58] were assayed using serum and commercial enzyme-linked immunosorbent assays (ELISAs), MyBioSource, San Diego, CA, USA [59,60,61]. Data were reported as pg/mL.

### 2.11. 16s rRNA Isolation and Sequencing and Microbiome Analysis

Microbiome analyses were performed in duplicate before and after intervention. A DNeasy 96 PowerSoil Pro QIAcube HT Kit (Germantown, MD, USA) [19,62] was used to isolate DNA from the portion of the fecal samples stored at −80 °C, and 250 mg of the sample was added to the Qiagen-supplied PowerBead tube and vortexed. A total of 60 mL of solution C1 (Qiagen lysing agent) was added, the tube was briefly vortexed manually, and then the tubes were secured to an adapter to be vortexed at maximum speed for 10 min. The tubes were then centrifuged at 1000× *g* for 30 s, and the supernatant was transferred to a 2 mL collection tube. A total of 250 mL of solution C2 (Qiagen precipitating agent) was added to the collection tubes, which were then vortexed for 5 s and then incubated on ice for 5 min. Tubes were then centrifuged for 60 s at 10,000× *g*, and 750 mL of supernatant is transferred to clean 2 mL collection tubes. A total of 200 mL of solution C3 (Qiagen precipitating agent) was added, and the tubes are briefly vortexed manually and incubated on ice for 5 min again. Following incubation, the tubes were centrifuged at 10,000× *g* for 60 s, 750 mL of supernatant was added to 2 mL collection tubes, 1200 mL solution C4 was added, and tubes were vortexed for 5 s. 675 mL of this solution was loaded into a Qiagen kit spin column and centrifuged at 10,000× *g* for one minute three times with flow-through being discarded between centrifugations. A total of 500 mL of solution C5 was added to the spin column, which was centrifuged at 10,000× *g* for 30 s and one minute with flow-through discarded between centrifugations. The spin columns were placed into clean 2 mL collection tubes, 100 mL nuclease-free water was added to the center of the spin column’s white filter membranes, and the samples were allowed to incubate at room temp for 5 min. Tubes were centrifuged for 30 s at 10,000× *g* to elute the DNA, spin columns were discarded, and samples were checked for DNA quantification with a Qubit fluorometric quantification meter on the broad range detection setting. DNA was fragmented and tagged with a 615f/806r adapter sequence before polymerase chain reaction amplification. Samples were sequenced using Nextera MiSeq (San Diego, CA, USA), and sequences were obtained from Illumina (San Diego, CA, USA) and Swift Biosciences (Ann Arbor, MI, USA) [62,63].

### 2.12. Data Management and Statistical Analysis

Categorical data was collected by a clinician employed at the Infectious Disease Clinic, ETSU. All data was recorded in an Excel spreadsheet and de-identified and shared via VPN with the primary investigators. The statistical analysis of samples was carried out on GraphPad Prism 9 (GraphPad Software, Inc., San Diego, CA, USA). Analyses were done utilizing Welch’s *t*-tests. Significant *p*-values are listed in individual figures. Standard deviation is shown in all figures. FFQ data was prepared via NutritionQuest by Block prior to statistical analysis, GraphPad Prism 9 analyses. The 16S sequencing data were processed using QIIME2-2021.11 [64]. Reads were binned into amplicon sequence variants (ASVs) using DADA2 via QIIME2 [65]. A phylogenetic tree was created using saté-enabled phylogenetic placement (SEPP). Taxonomy was assigned using a naïve-Bayes classifier [66] trained on the SILVA [67] 16S database provided in QIIME2-2021.11. For alpha diversity, samples were rarefied to 52,495 reads (the sequencing depth of the least sequenced sample) for diversity analyses. Faith’s phylogenetic diversity was used to assess alpha diversity, and unweighted UniFrac distance was used to assess community composition (beta diversity). Unweighted UniFrac was used as a distance matrix in beta diversity, using the pseudo-*F* statistic (PERMANOVA) for significance. Rare ASVs with less than 100 total reads across all samples were removed for analysis with Gneiss and SCNIC [68,69]. Gneiss was used to perform hierarchical clustering on ASVs and to create isometric log-ratio balances from the hierarchical clustering tree. A Kruskal–Wallis test was used to compare balances across HIV status. The Kruskal–Wallis test was used due to non-normality and outliers. SCNIC (with SPARCC correlation) [69,70,71] was used to create a co-occurrence network of ASVs, and a shared minimum distance of 0.35 was used to cluster ASVs into highly co-occurring modules. ASV relative abundances were summed within modules. Differential abundance analysis using ANCOM was performed on modules, as well as at the phylum and genus levels to identify differentially abundant taxa based on HIV status.

## 3. Results

### 3.1. Recruitment, Anthropometrics, Liver Steatosis and Fibrosis, and HARRT

**Figure 1:**
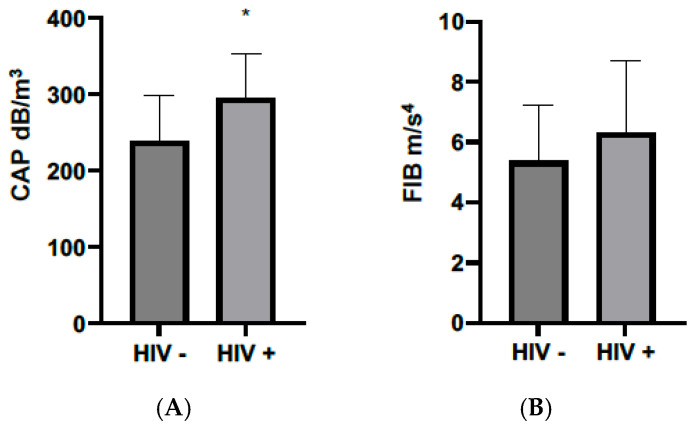
Measures of liver health. (**A**) Hepatic steatosis (CAP) and (**B**) hepatic fibrosis. (FIB) FibroScan. Unpaired Welch’s *t*-tests: CAP (*p* = 0.005); fibrosis (*p* = 0.181) * *p* < 0.05 for significance.

This study examined a cohort of 19 HIV-positive men on HAART and 21 male controls without HIV (Table 2). HIV-positive males were recruited through East Tennessee State University’s Infectious Disease Specialty Clinic (IDSC), Johnson City, Tennessee. Male controls were recruited via convenience recruiting to assist in matching for age and BMI. All subjects had their anthropometrics measured, blood draw, and liver scans performed at the IDSC (Table 2). Subjects completed a Block Food Frequency Questionnaire (FFQ), which they returned to the IDSC along with a full stool sample. Subjects were deidentified and data was shared via a virtual private network (VPN). Although all efforts were made to recruit males of similar age, a significant difference was found between groups, with the HIV-positive males being older when compared to controls (*p* < 0.045). HIV-positive males had a significantly higher hip-to-waist ratio when compared to controls (*p* < 0.001), indicating a significant difference in body fat distribution between the groups. Additionally, HIV-positive males had a significantly higher CAP score when compared to male controls (*p* < 0.005) (Figure 1A), indicating metabolic abnormalities resulting in liver steatosis, and nonsignificant differences in liver fibrosis (*p* = 0.181) (Figure 1B). Medications that the HIV-positive males were taking at the time of sample collection are shown in Table 3.

### 3.2. Food Frequency Questionnaire Data and Fecal Proximate Analyses

When examining the 1-month FFQ data, there was no significant difference found between groups in total kcal intake, percent macronutrients, or fiber grams (Table 4). When examining consumption of fruit and vegetable servings (cups), there was no significant difference between groups (Table 4).

The proximate analysis of the stool samples was completed to compare differences in non-digested nutrients, stool moisture, and total dry matter—indicators of malabsorption. There were no significant differences found between groups (Table 4).

### 3.3. Fecal Fermentation

**Figure 2:**
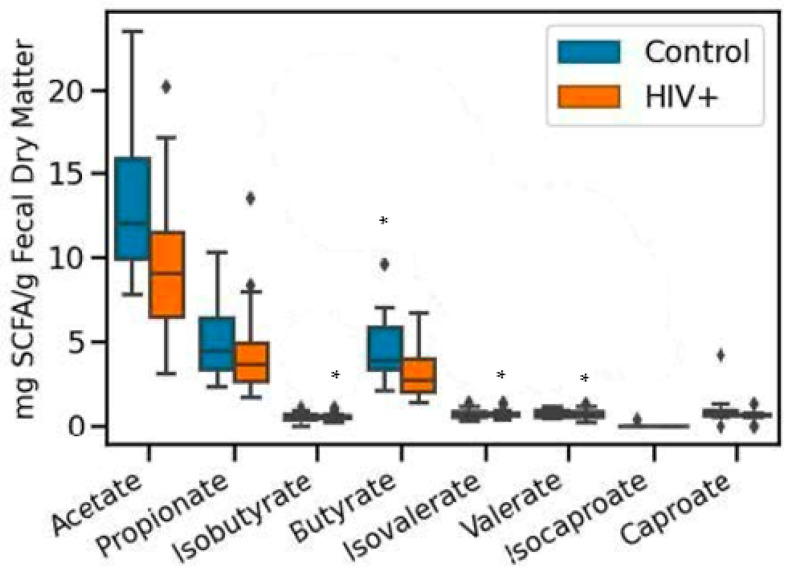
SCFAs (mg) per gram of dry fecal matter. Isobutyrate (*p* = 0.002), butyrate (*p* = 0.001), isovalerate (*p* = 0.001), valerate (*p* = 0.033), and acetate/propionate/butyrate (APB combined) (*p* = 0.046) * (*p* < 0.05) for significance. ◆ Outlier.

The analysis of the fecal fermentation profile of the stool samples showed distinct significant differences between groups (Table 5 and Figure 2). The HIV-positive males had significantly lower levels of straight-chain SCFA butyrate (*p* < 0.001), and the major SCFAs—acetate, propionate, and butyrate—combined (*p* < 0.005), when compared to males without HIV. Males without HIV had significantly lower levels of straight-chain SCFA valerate (*p* < 0.033), as well as branched-chain SCFAs isovalerate (*p* < 0.001) and isobutyrate (*p* < 0.002). Isocaproate was only detected in two subjects in the HIV-negative group (0.102% ± 0.386%).

**Figure 3:**
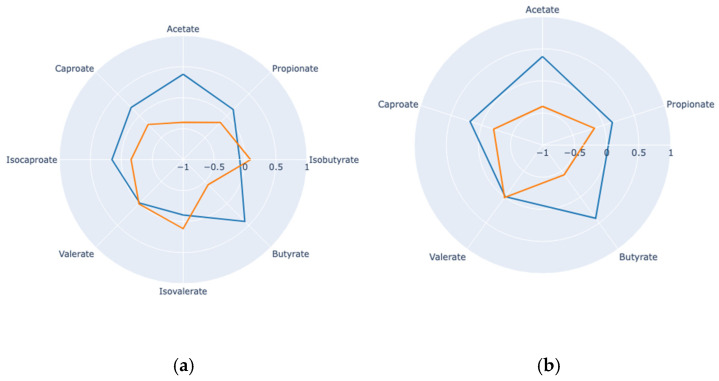
Fecal short-chain fatty acid Z-scores. (**a**) Predominant SCFA and (**b**) straight-chain fatty acids. The orange line indicates HIV-positive males, and the blue line indicates male controls without HIV.

Z-scores were performed for SCFAs to visualize the distributions between groups (Figure 3a). HIV-positive males had notably smaller SCFA production when compared to males without HIV, with the exception of two branched short-chain fatty acids, isobutyrate and isovalerate (Figure 3b).

### 3.4. Intestinal Permeability

Measures of intestinal permeability were analyzed via ELISA using serum collected from subjects at the time of recruitment. There were no significant differences between the groups in all three measures (Table 6). When controlled for BMI as well as age, there were no significant interactions between the groups. Flagellin, a measure of intestinal permeability; and claudin-21 and I-FABP, measures of jap junction integrity were similar between the groups, with I-FABP exhibiting non-significant higher levels in HIV-positive males. Additional analyses of intestinal permeability measures controlling for BMI and age showed no significant differences between groups.

### 3.5. 16s rRNA Gene Sequencing

**Figure 4:**
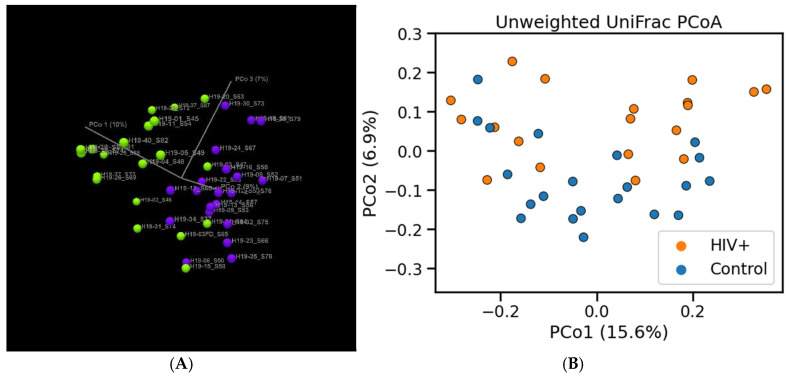
(**A**) b-diversity, Principal Coordinate Analysis (PCoA) with three axes (3 dimensional) to capture distribution of community differences between groups; green dots represent HIV+ males and purple dots, HIV− males illustrating the spread of each individual subject based on HIV status (**B**) Significant difference in b-diversity (community composition) by HIV status; unweighted UniFrac PCoA (*p* < 0.006) *p* < 0.5 set at level of significance. PCo1 (15.6%) captures the overarching pattern of community differences between groups. PC02 (6.9%) captures a secondary pattern of community differences between groups. Unweighted UniFrac removes abundance and allows focus on the presence/absence of taxa.

**Figure 5:**
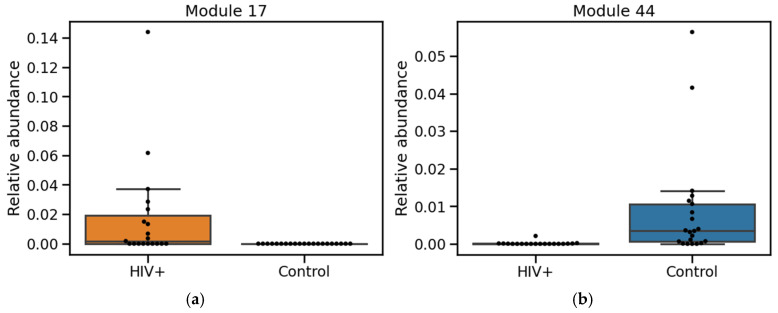
Two significantly different modules, based on ANCOM (analysis of communities of microbiomes): (**a**) #17 *Prevotella* is higher in relative abundance in HIV+ males. (**b**) #44 *Lachnospiraceae* is higher in relative abundance in HIV-males.

We performed 16s rRNA gene sequencing on participant fecal samples. There were no significant differences within the HIV-positive males’ alpha-diversity (*p* = 0.61). Consistent with other research findings, we found significant differences between HIV-positive males and male controls without HIV illustrated by the divergent beta diversity (Figure 4A). The phylogenetic community composition (unweighted UniFrac PCoA) (Figure 4B) is significantly different when comparing HIV-positive versus controls without HIV (F = 1.83, *p* = 0.006). PERMANOVA analysis exhibited significant difference in β-diversity between the groups (*p* < 0.00001) (Table 7). Two significantly different modules, based on ANCOM (analysis of communities of microbiomes): #17 *Prevotella* (Figure 5a) is higher in relative abundance in HIV+ males. #44 *Lachnospiraceae* (Figure 5b) is higher in relative abundance in HIV-males.

**Figure 6:**
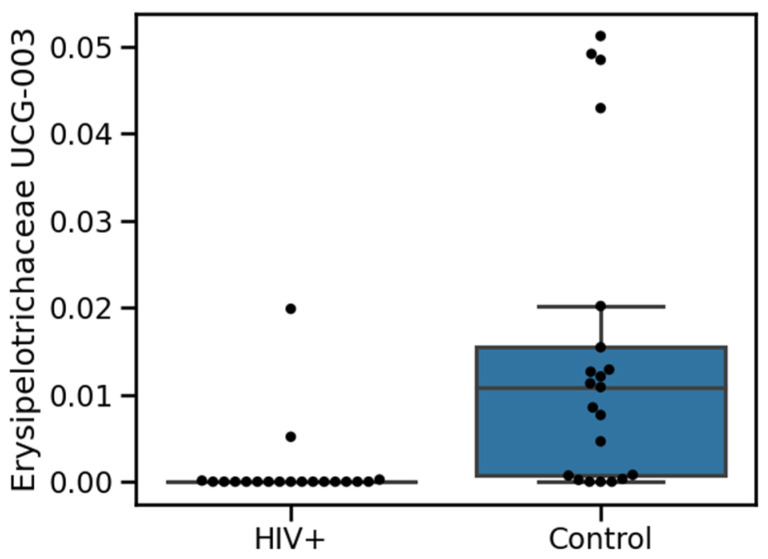
Genus *Erysipelatoclostridiaceae UCG-003* decreased in HIV-positive males.

Differential abundance analysis identified a significantly decreased abundance of the genus *Erysipelatoclostridiaceae UCG-003* in HIV-positive males (ANCOM W = 285/300) (Figure 6).

**Figure 7:**
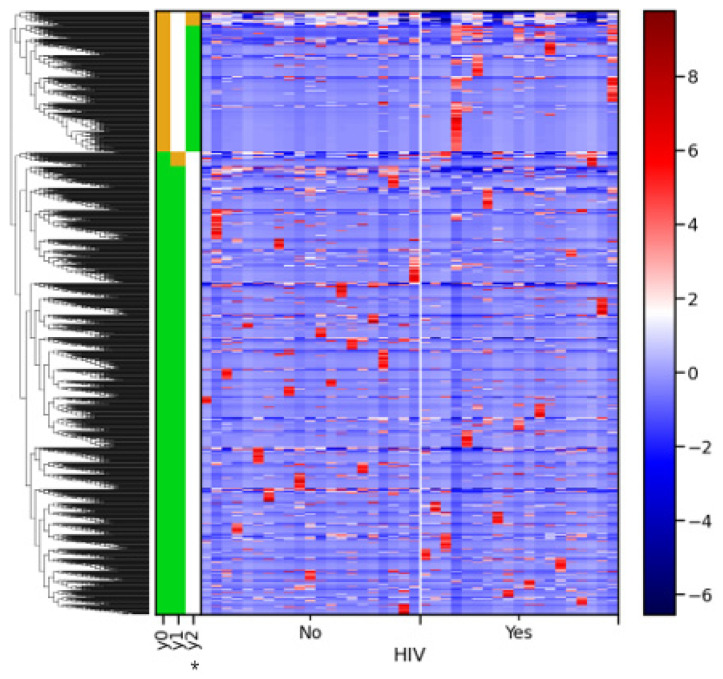
Heat map co-occurrence based clustering in qiime gneiss. Heat map of Kruskal-Wallace one-way ANOVA of taxa in males with and without HIV. Significant difference in y2 (*p* < 0.001) (indicated by green color in the y2 column compared to the orange and white colors in y0 and y1 columns). * *p* < 0.05.

**Figure 8:**
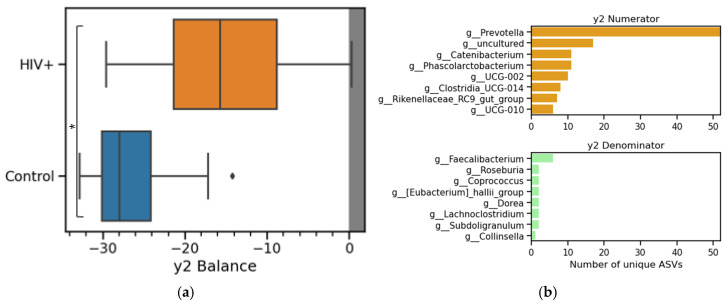
(**a**) Box and whisker plot and y2 Balance, (**b**) Genus Rank y2 balance (*p* < 0.001), Kruskal–Wallis test. * *p* < 0.05. HIV positive males had a significantly different number of amplicon sequence variants (ASV), indicating a significant difference in microbial communities between groups. Specific communities that were identified are shown in figure (**b**). ◆ Outlier.

Assessing isometric log-ratios within a tree built from hierarchical co-occurrence-based clustering, a method suited for compositional data, revealed a significant association between one “balance” (log-ratio between many taxa) and HIV status, as illustrated in the heat map (Figure 7). The balance “y2” was higher in males with HIV (*p* < 0.001; Kruskal–Wallis test) (Figure 8a). The “y2” balance consists of a ratio of (A) a group of co-occurring taxa dominated by Prevotella to (B) a group of co-occurring taxa including the butyrate-producing genera *Faecalibacterium*, *Roseburia*, *Coprococcus*, *Eubacterium*, *Dorea*, *Lachnoclostridium*, *subdoligranulum*, and *Collinsella* (Figure 8b). These findings suggest that in individuals with HIV, there is a relative enrichment in *Prevotella* and related taxa, and/or a decrease in the aforementioned butyrate-producing genera. These findings are consistent with other investigations in populations with HIV.

**Figure 9:**
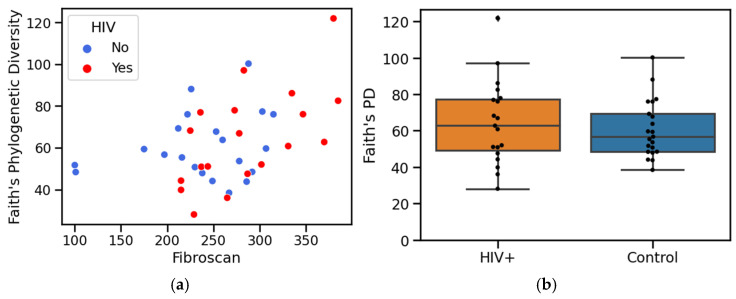
α-diversity controlled for liver steatosis and non-controlled comparisons of males with and without HIV. (**a**) Faith’s Phylogenic Diversity controlled for liver steatosis: (*p* < 0.048); (**b**) Faith’s Phylogenic Diversity non-controlled for liver steatosis (*p* < 0.057) (*p* < 0.05 for significance). ◆ Outlier.

In analyses of alpha diversity as a function of HIV and liver steatosis (CAP), we saw a significant decrease in alpha diversity when CAP was held constant between the groups; controls with and without HIV (*p* > 0.048) (Figure 9a). However, when CAP was not controlled, an increase in alpha diversity among males with HIV approached significance (*p* > 0.057) (Figure 9b). These findings indicate that there is an increased alpha diversity that is associated with fatty liver in HIV, possibly indicating metabolic health differences between the groups. There were no significant findings when controlling for fibrosis, HIV, and alpha diversity (*p* < 0.098). There were no significant interactions observed between other anthropometric measures, BMI and hip-to-waist ratio and liver scans in males with HIV.

## 4. Discussion

This study attempts to define changes in gut permeability, the intestinal microbiome, fecal fermentation profile, and lipodystrophy in HIV-positive males on HAART, while also examining dietary intake and the macronutrient content of stool. When exploring measures of gut permeability, our findings did not support it as an influential factor. However, additional measures of LPS or LBP could have been considered as a complement, since other investigators have shown associations among inflammatory markers, LPS, and LBP [11,12,19].

When examining microbial differences, we found an increase in relative abundance of *Prevotella* and *Lachnospiraceae*, and a decrease in *Faecalibacterium*, *Roseburia*, *Coprococcus*, *Eubacterium*, *Dorea*, *Lachnoclostridium*, *subdoligranulum*, and *Collinsella*. Our findings are similar to McHardy et al. who found similar changes in decreases in butyrate-producing bacteria in males with HIV that were not currently on HAART; decreases in *Roseburia*, *Coprococcus*, *Eubacterium*, *Alistipes*, *Ruminococcus* and *Lachnospira* [75]. Changes in microbial diversity and abundance in males with HIV on HAART does not appear to be related to pharmaceutical interventions, as found in McHardy et al. investigations [75]. In an SIV (simian immunodeficiency virus) infection model, Blum et al. were able to partially restore microbial eubiosis with antiretroviral therapy, but not achieve full recovery with pharmaceutical interventions [76]. Armstrong et al. found a strong correlation between LBP and a decrease in beneficial butyrate-producing microbes [76]. These findings were consistent with MSM with or without HIV, and with or without HAART [77]. At the time of sample collection, our study revealed diverse pharmaceutical treatments in our males with HIV. Due to our small sample size and variability in medical treatment, we could not control for this variable. Our reasoning infers that the distinct microbial differences between groups may be related to factors outside of the scope of this study.

To further explore microbiome and fecal fermentation profiles in the current study, distinct differences in microbial relative abundances were observed between males with an without HIV. Not only did the males with HIV have an increased abundance of *Prevotella* and *Lachnospiraceae*, but they also exhibited significantly lower amounts of butyrate, indicating a shift towards a more pro-inflammatory profile. Not only were there differences in SCFAs, but males with HIV also had significantly higher levels of the BCFAs (isobutyrate, isovalerate, and valerate), compared to males without HIV.

There were no significant differences in dietary intake of macronutrients or macronutrient content of the fecal dry matter. These findings suggest increased BCAA metabolism in the large intestine, consistent with a more pro-inflammatory metabolic profile. Aguirre et al. found an increase in BCFAs in high-protein diets and an increase in pH in the distal colon [78]. There was a decrease in distal colon pH with the high-carbohydrate diet, which has been suggested to inhibit protein fermentation in the intestine [78]. Van Nuenen et al. found similar results when introducing *Clostridium difficile* in an in vitro model of the proximal colon; an increase in protein fermentation metabolites, such as ammonia, isobutyrate, and isovalerate [79]. It is possible that the changes observed in the relative abundance of *Prevotella* among subjects with HIV include species that are more proteolytic and promote a higher pH in the colon.

Differences in the fermentation profiles of our males with HIV may have contributed to the higher H:W and NAFLD measures we observed. Chen et al. found that commercial Duroc pigs with a significantly higher abundance of *Prevotella copri* had significantly greater fat accumulation and were correlated with obesity-related serum markers, including LPS, BCAAs, and aromatic amino acids [80]. Chen et al. also found an increase in gut permeability and chronic inflammation [80]. When germ-free mice were galvanized with *Prevotella copri*, it was found that fat deposition increased significantly, and chronic inflammatory responses were initiated via TLR4 and mTOR signaling pathways [80]. This increase in gene expression resulted in lipogenesis and fat accumulation, as well as an inhibition of lipolysis and muscle growth [80]. Interestingly, Chen et al. also found that fat accumulation in germ-free mice was higher when *Prevotella copri* infected mice were fed a high fat diet vs. a high fiber and regular chow diets, indicating that *Prevotella copri* may influence fat metabolism regardless of diet [80]. These findings shed light on differences found between males with and without HIV. These findings are similar to other research that has found NAFLD and insulin resistance to co-occur with the *Prevotella*-rich enterotype [7,9,19,77,81,82].

In addition to increases in *Prevotella*, we found increases in the relative abundance of *Lachnospiraceae*. The *Lachnospiraceae* family belongs to the *Clostridiam* cluster *XIVa* of the phylum Firmicutes, one of the primary butyrate-producing microbes [39,40,82]. Bacteria, *Blautia*, *Coprococcus*, *Dorea*, *Lachnospira*, *Oribacterium*, *Roseburia*, and *L-Ruminococcus*, are also found within this family and were found to be decreased in males with HIV [82]. Interestingly, *Lachnospiraceae* has been shown to increase in abundance with aging and in certain disease conditions. Diseases associated with an increase in *Lachnospiraceae*, include DM2 and liver disease [82,83]. Correlations have been established between a high abundance of *Lachnospiraceae* and disturbances in lipid and glucose metabolism and DM2 [83]. Shen et al. found an increased abundance of *Lachnospiraceae* to be a primary contributor to the development and progression of NAFLD [84]. Lastly, *Erysipelatoclostridiaceae UCG-003* has been found to be enriched in obesity and inflammatory conditions and positively associated with insulin resistance due to saccharide and protein metabolism [85]. Overall, males with HIV exhibited a more pro-inflammatory, insulin-resistant enterotype that would support the development and progression of NAFLD [11,12,13,14,77]. Additionally, anthropometric differences were observed in relation to the microbiome. Our findings supported findings in the literature, in which a pro-inflammatory enterotype is associated with altered metabolism, resulting in lipodystrophy and android obesity [14,85]. Our males with HIV had a significantly higher hip-to-waist ratio, indicating visceral body fat distribution, along with significantly higher liver steatosis when compared to males without HIV. The non-significant difference in dietary intake may be attributable to regional differences. Thus, differences in body composition, liver steatosis, and the intestinal microbiome may not be overly influenced by diet alone, but rather by differences in fermentation metabolites produced in the gut.

A notable limitation of our study was the significant age difference between groups, with HIV-positive men being older than controls (42.00 ± 11.26 vs. 48.53 ± 7.94; *p* < 0.045), a possible consequence of the small sample size that may have contributed to some of the anthropometric differences seen between groups. According to Hunter et al., visceral fat accumulates over multiple decades, peaking during the 5th–7th decade of life in males [86]. Although there is a significant age difference between groups, the difference in fat distribution is most likely not attributable to age alone. An additional limitation of this study may be the differences in medications and types of comorbidities within the group with HIV. Only one subject was taking a medication that has been associated with ER dysfunction, protein misfolding, and lipodystrophy. All other males with HIV were taking newer generations of medications that have not been shown to have similar metabolic side effects. However, five subjects were taking medications for hyperglycemia, and it is known that individuals with altered glucose metabolism (diabetes mellitus) present with a pro-inflammatory enterotype. We did not control for any other metabolic conditions other than those that would impact nutrient absorption, such as inflammatory bowel disease, celiac disease, irritable bowel syndrome, and other autoimmune diseases. An additional limitation of this study is the sample size. Some of our confounding factors, such as a significant age difference, although just within the level of significance, may have contributed to the differences we observed between groups. Given the limitations of this study, caution is warranted in interpreting the results. Future areas of research include controlling for lifestyle factors, such as sexual practices and the co-occurrence of STIs. Long-term investigations could utilize interventions to increase beneficial SCFAs, particularly butyrate. These findings lay the groundwork for investigations into factors related to the intestinal microbiome and the role of SCFAs in lipodystrophy among males with HIV. Our goal is to better understand the impact of lifestyle on the microbiome and fecal fermentation profile in MSM, and to explore potential therapeutic interventions targeting the microbiome and chronic inflammation that impact overall health outcomes in this population.

## 5. Conclusions

There is an intriguing association between HIV positivity, changes in the microbiome and fecal fermentation profiles, and measures of lipodystrophy. Our study found significant differences in the relative abundances of *Prevotella* and *Lachnospiraceae*, with *Prevotella* and *Lachnospiraceae* being increased in males with HIV. Additionally, we observed significant differences in fermentation products: males with HIV had lower butyrate and higher BCFAs (isobutyrate, isovalerate, and valerate) compared to males without HIV, indicating a more pro-inflammatory fecal fermentation profile. Lastly, we demonstrated that males with HIV had a significantly higher H:W and steatosis of the liver, when compared to HIV-negative controls. Overall, these findings suggest potential associations among the microbiome, fermentation products, and lipodystrophy, regardless of diet and absorption, or HAART.

## Figures and Tables

**Table 1:** Enterotypes.

Condition	Increased Abundance	Decreased Abundance
Plant-Based Diet ^1^	*Prevotella*	*Bacteroides*
Western Diet ^2^	*Bacteroides*	*Prevotella*
Obesity ^3^	Firmicutes, *Prevotella copri*	*Bacteroides*, *Faecalibacterium praznitzii*
Obesity Bacteroides 2 (Bact2) ^4^	*Bacteroides*	
IBD/IBS ^5^	Firmicutes (*Ruminococcus*)	*Bacteroides*
Autism/Neuro ^6^	Firmicutes	*Bacteroides*
HIV ^7^	*Prevotella copri*	*Bacteroides*, *Faecalibacterium praznitzii*

^1,2,6^ [20], ^3^ [32], ^4^ [33], ^5^ [23], ^7^ [19].

**Table 2:** Clinical parameters.

Clinical Parameters	HIV Negative	HIV Positive	*p* Value *
Sample Size	21	19	
Age (years)	42.00 ± 11.26	48.53 ± 7.94	*p* = 0.045 *
BMI (kg/m^2^) ^1^	26.61 ± 3.46	28.70 ± 4.01	*p* = 0.096
Hip-to-Waist Ratio ^2^	0.87 ± 0.06	0.94 ± 0.08	*p* = 0.001 *
Race	1 African American 1 Caucasian	3 African Americans 15 Caucasians 1 Latino	N/A
Gender	Male	Male	N/A
Liver Steatosis (CAP) dB/m ^3^	238.81 ± 59.57	294.74 ± 57.96	*p* = 0.005 *
Liver Fibrosis (FIB) m/s ^4^	5.36 ± 1.89	6.3 ± 2.4	*p* = 0.181

* *p* < 0.05 significance; ^1^ BMI: <18.5 underweight; 18.5–24.9 healthy weight; 25.0–29.9 overweight; >30 obese [45]. ^2^ Hip-to-waist Ratio: ≥0.85 women; ≥0.90 men [45,72]. ^3^ Controlled attenuation parameter (CAP) decibel per meter (dB/m). The grades are assigned as follows: S0, no steatosis (0–10% fat; 0–237 dB/m); S1, mild steatosis (11–33% fat; 238–259 dB/m); S2, moderate steatosis (34–66% fat; 260–292 dB/m); and S3, severe steatosis (>67% fat; ≥293 dB/m) [73]. ^4^ Acoustic Radiation Force Impulse (ARFI) reference values: 1.35 m/s—absent or mild fibrosis (F0 or F1); 1.35–1.55 m/s—significant fibrosis (F2); 1.55–1.80 m/s—severe fibrosis (F3); >1.80 m/s—cirrhosis (F4) [74].

**Table 3:** Medications of recruited HIV-positive males.

Subject	Medications
1	Calcium 600, Claritin, Descovy, Finasteride, Prezista, Ritonavir, Rosuvastatin, Calcium, valacyclovir HCl, Vitamin D (Ergocalciferol)
2	Atorvastatin Calcium, Descovy, Intelence, Isentress, Levoxyl, Prezista, Quetiapine Fumarate, ranitidine HCl, Ritonavir, Sertraline HCl, Sildenafil Suspension Oral, Zidovudine
3	Amcinonide, Atorvastatin, D3-1000, Multi for him 50+, ranitidine HCl, Triumeq
4	Aspirin, Descovy, Imiquimod, Loratadine, Pravastatin, Ramipril, Tamsulosin, Tivicay
5	Aspirin, Benadryl, Escitalopram, Fenofibrate, Fish Oil, Flonase, Genvoya, Ibuprofen, Isosorbide Mononitrate 30 MG, Isosorbide Mononitrate 60 MG, Lisinopril, Mirtazapine, Multi-Vit, Nitroglycerine, Pravastatin, trazadone HCl, Vit C, Zyrtec
10	amlodipine, Descovy, Ezetimibe, Fenofibrate, Indapamide, Jardiance, Lipitor, Lisinopril, Metformin, Pioglitazone, Testosterone, Tivicay, Vit D,
11	Abacavir, Aripiprazole, Escitalopram Oxalate, Freestyle Libre, Glucagon Emergency, Humalog, Levothyroxine Sodium, Lisinopril, Ondansetron, Tivicay, Vit-D
15	Aspirin, Benadryl, Descovy, Isentress, Lipitor, lisinopril, Metoprolol, Nitroglycerine
21	Aspirin, Descovy, Escitalopram Oxalate, Fluconazole, Lorazepam, Losartan Potassium, Mometasone Furoate, Montelukast Sodium, Mucinex, ProAir HFA, Ranitidine, Rosuvastatin Calcium, Tivicay, Triamcinolone Acetonide, Valcyclovir, Vitamins To Go Men
25	Atorvastatin Calcium, Centrum Men, Genvoya, Spiriva Respimat, Vitamin D
26	Aspirin, Cyclobenzaprine HCL, Descovy, Dicyclomine HCL, Flunisolide, Lamotrigine, Lisinopril, Melatonin, Metformin, Miconazole Nitrate, Naproxen, Nystatin, Omeprazole, Pravastatin Sodium, Pristiq, Sildenafil Citrate, Tivicay, Vitamin D, Zolpidem Tartate
27	Albuterol, Anusol-HC, Aripiprazole, Aspirin, Biotin, Contour Next, Descovy, Diltiazem, Duloxetine HCL, Emgality, Ferrous Sulfate, Flonase, Fluconazole, Isentress, Losartan Potassium, Myrbetriq, Nexium, Nucynta, Ondansetron, Oxycontin, Phentermine HCL, Pioglitazone HCL ER, Pyridium, Rosuvastatin Calcium, Singulair, Sumatriptan Succinate, Tizanidine, Trazodone HCL, Trulicity, Vitamin D-3
28	Abacavir Sulfate-lamivudine, Aspirin, BD Insulin Syr, Contour Next, Easy Touch Insulin Syr, Edurant, Escitalopram Oxalate, Fosinopril Sodium, Lantus, Micro Lancets, Novolog, Pen Needles, Rosuvastatin Calcium, Victoza, Vitamin D, Zyrtec
29	Aspirin, Biotin, Escitalopram Oxalate, Famotidine, Genvoya, Ibuprofen, Multivitamins, Pravastatin Sodium, Testosterone, Vitamin B-12, Vitamin E
31	Benadryl, Biktarvy, CVS Vitamin D3, Duloxetine, Hydroxyzine HCL, Mirtazapine, Modafinil, Multivitamin Men, Nexium, Probiotic, Valacyclovir
37	Beconase AQ, Calcium, Fish Oil, Gemfibrozil, Intelence, Isentress, Lamivudine-Zidovudine, Meloxicam, Omeprazole, Prezista, Ritonavir
38	Atorvastatin Calcium, Biktarvy, Clonazepam, Lisinopril-hydrochlorothiazide, Montelukast Sodium, Valcyclovir HCL, Vitamin D, Zyrtec
39	Aripiprazole, Armodafinil, Atorvastatin Calcium, Biktarvy, Tamsulosin HCL, Vitamin D3
40	Biktarvy

Triumeq© (abacavir, dolutegravir, Iamivudine); Descovy© (emtricitabine and tenofovir alafenamide); Genvoya© (elvitegravir, cobicistat (P450 inhibitor), emtricitabine, and tenofovir alafenamide); Biktarvy© (bictegravir, emtricitabine and tenofovir alafenamide); Isentress© (raltegravir); Prezista© (darunavir); Intelence© (etravirine); Tivicay© (dolutegravir).

**Table 4:** FFQ and proximate analyses of stool samples.

	HIV Negative	HIV Positive	*p* Value *
Sample Size	21	19	
FFQ			
Total Energy intake (kcals)	2160.87 ± 699.53	2418.33 ± 1095.60	*p* = 0.388
% Fat Intake	37.29 ± 6.50	36.07 ± 6.86	*p* = 0.569
% Sat Fat Intake	12.76 ± 2.80	11.82 ± 2.65	*p* = 0.285
% Protein Intake	15.42 ± 2.24	14.26 ± 3.47	*p* = 0.222
% Carbohydrate Intake	44.27 ± 10.14	47.71 ± 9.68	*p* = 0.279
% Alcohol Intake	6.06 ± 5.74	4.07 ± 8.46	*p* = 0.397
Fiber Intake (grams)	21.12 ± 11.74	19.55 ± 15.20	*p* = 0.719
Servings of Fruit (cups)	1.34 ± 1.24	1.37 ± 1.57	*p* = 0.953
Servings of Vegetables (cups)	1.86 ± 0.95	1.53 ± 1.16	*p* = 0.328
Proximate Analyses of Stool			
Bomb Calorimetry (kcal)	5302.93 ± 477.60	5457.18 ± 415.43	*p* = 0.282
Soxhlet (% crude lipids)	26.66 ± 0.11	23.49 ± 0.11	*p* = 0.372
Kjeldhal (% crude protein)	30.42 ± 0.10	28.91 ± 0.05	*p* = 0.534
% Insoluble Dietary Fiber (IDF)	35.27 ± 0.10	39.01 ± 0.14	*p* = 0.354
% Soluble Dietary Fiber (SDF)	15.01 ± 0.09	15.52 ± 0.10	*p* = 0.870
% Total Dietary Fiber (TDF)	50.29 ± 0.14	54.51 ± 0.21	*p* = 0.468

* *p* < 0.05 significance; values expressed as mean (standard deviation).

**Table 5:** Short-chain fatty acids in stool samples.

	HIV Negative	HIV Positive	*p* Value *
Sample Size	21	19	
SCFAs (%AUC) †			
Acetate	30.89 ± 4.12	29.10 ± 6.48	*p* = 0.313
Propionate	23.52 ± 3.35	24.37 ± 5.95	*p* = 0.585
Isobutyrate	27.66 ± 0.05	28.73 ± 0.08	*p* = 0.002 *
Butyrate	29.48 ± 3.82	24.97 ± 4.38	*p* = 0.001 *
Isovalerate	5.79 ± 1.79	8.61 ± 2.83	*p* = 0.001 *
Valerate	4.99 ± 1.53	6.37 ± 2.25	*p* = 0.033 *
Isocaproate #	n/a	0.102 ± 0.386 (*n* = 2) #	n/a
Caproate	1.94 ± 1.86	1.32 ± 0.96	*p* = 0.191
Acetate + Propionate + Butyrate (APB)	83.88 ± 4.25	78.44 ± 6.60	*p* = 0.0046 *

† Percent area under the curve (% AUC); * *p* < 0.05 significance, values expressed as mean (standard deviation); # Isocaproate was detected in two HIV− individuals (0.102% ± 0.386%) and was absent in HIV+ individuals.

**Table 6:** Intestinal permeability measures in serum.

	HIV Negative	HIV Positive	*p* Value *
Sample Size	21	19	
Flagellin (ng/mL) *	0.9366 ± 0.1581	1.0450 ± 0.1084	*p* = 0.497
Claudin-21 (ng/mL) *	2317 ± 501.6	2433 ± 116.0	*p* = 0.818
IFABP (pg/mL) *	183.0 ± 49.59	254.7 ± 71.74	*p* = 0.156
Flagellin (ng/mL) **	m = 0.8525 IQR = 0.7290–0.9274	m = 0.9704 IQR = 0.6273–0.5741	*p* = 0.598
Claudin-21 (ng/mL) **	m = 1404.6 IQR = 943.7–4285.9	m = 1995.7 IQR = 1213.4–4173.2	*p* = 0.425
IFABP (pg/mL) **	m = 137.52 IQR = 79.60–254.39	m = 220.37 IQR = 122.80–372.96	*p* = 0.679

* *p* < 0.05 significance; values expressed as mean (standard deviation); ** *p* < 0.05 significance; values expressed as median and Interquartile Range (I)QR 1–3; Mann-Whitney U.

**Table 7:** PERMANOVA analysis (Bray–Curtis).

Variable	Groups	Pseudo-f Statistic	*p*-Value *	
Type	1, 2	2.75139	0.00001 *	
Group 1	Group 2	Pseudo-f statistic	*p*-value	*p*-value (Bonferroni)
1	2	2.75139	0.00001 *	0.00001

Group 1: HIV-positive males; Group 2: males without HIV; * *p* < 0.05 set at level of significance.

## Data Availability

The raw data for microbiome sequencing can be found at NCBI; SRA #PRJNA1490905. The raw data supporting the conclusions of this article will be made available by the authors upon reasonable request.

## Supplementary Materials

The following supporting information can be downloaded at: https://www.mdpi.com/article/10.3390/nu18142328/s1, File S1: Informed Consent, File S2: Block food frequency questionnaire.

## Abbreviations

The following abbreviations are used in this manuscript:

17A^-/-^	Interleukin 17A Knock-out mouse
H:W	Hip-to-waist ratio
AIDS	Acquired Immunodeficiency Syndrome
ANCOM	Analysis of Composition of Microbiomes
APB	Acetate/Propionate/Butyrate
ARFI	Acoustic radiation force impulse
ASV	Amplicon sequence variant
BACT2	Bacteroides 2 enterotype
BCAAs	Branched chain amino acids
BCFAs	Branched chain fatty acids
BMDCs	Bone marrow derived dendritic cells
BMI	Body mass index
C	Carbon
CAP	Controlled Attenuation Parameter
CCR5	Chemokine receptor 5
CD4	Cluster of differentiation 4 (T cells)
CD8	Cluster of differentiation 8 (T cells)
CDC	Center of Disease Control
CRP	C-Reactive Protein
CVD	Cardiovascular disease
DDW	Deionized distilled water
DFDS	Dietary Fiber Data Sheet
DM2	Diabetes Mellitus type 2
DNA	Deoxyribonucleotide acid
DW	Dry weight
ELISA	Enzyme-linked Immunoassay
ER	Endoplasmic Reticulum
FFA	Free Fatty Acid
FFAR	Free Fatty Acid Receptor
FFQ	Food Frequency Questionnaire
FI	Fusion Inhibitor
Foxp3	Forkhead box P3
GC	Gas Chromatography
GF	Germ free
GPCR	G protein-coupled receptor
HAART	Highly active antiretroviral therapy
HAT	Histone acetyltransferase
Hep B	Hepatitis B virus
Hep C	Hepatitis A virus
HCl	Hydrochloric acid
HDAC	Histone deacetyltransferase
HgO	Mercury III oxide
HIF	Hypoxia-inducible Factor
HIV	Human immunodeficiency virus
HFD	High-fat diet
HPV	Human papillomavirus
HTN	Hypertension
IBD	Inflammatory bowel disease
IDF	Insoluble dietary fiber
IDSC	Infectious Disease Specialty Clinic
IκB	Nuclear factor in B-cell inhibition
IFABP	Intestinal Fatty Acid Binding Protein
IFNγ	Interferon gamma
II	Integrase Inhibitor
IL-1β	Interleukin 1 beta
IL-6	Interleukin 6
IL-10	Interleukin 10
IL-17	Interleukin 17
IL-18	Interleukin 18
IL-23	Interleukin 23
K_2_SO_4_	Potassium sulfate
LDL-C	Low Density Lipoprotein C
LFD	Low-fat diet
LPS	Lipopolysaccharide
LPB	Lipopolysaccharide Binding Protein
MES-TRIS	2-(*N*-morpholino)ethanesulfonic acid—tris(hydroxymethyl(amino methane
mdDCs	Monocyte-derived dendritic cells
MSM	Men that have Sex with Men
mTOR	Mammalian target of rapamycin
NaCO_3_	Sodium carbonate
Na_2_O_3_S_2_	Sodium thiosulfate
Na_2_SO_4_	Sodium sulfate
NaOH	Sodium hydroxide
NAFLD	Non-alcoholic fatty liver disease
NF-κB	Nuclear Factor-kappa B
NRTI	Nucleoside Reverse Transcriptase Inhibitor
NNRTI	Non-Nucleoside Reverse Transcriptase Inhibitor
RA	Rheumatoid Arthritis
rRNA	Ribosomal ribonucleic acid
OTU	Operational Taxonomic Unit
PA	Proximate Analysis
PCoA	Principle Coordinates Analysis
PERMANOVA	Permutational multivariate analysis of variance
PET	Petroleum
pH	Potential of hydrogen
PI	Protease Inhibitor
Pol gamma	DNA polymerase gamma
PPAR-γ	Peroxisome proliferator-activated receptor gamma
PrEP	Pre-exposure prophylaxis
RAI	Rectal anal intercourse
SAD	Standard American diet
SCFA	Short-Chain Fatty Acid
SCNIC	Sparse correlation network investigation for compositional data
SDF	Soluble dietary fifer
SIV	Simian immunodeficiency virus
SparCC	Sparse compositional correlation
SPF	Specific-pathogen free
STI	Sexually transmitted infection
Tc	Cytotoxic T-cell
TDF	Total dietary fiber
TGF-β	Transforming growth factor beta
Th	Helper T-cell
TJ	Tight junction
TLR	Toll-like receptor
Treg	Regulator T-cell
WAT	White adipose tissue
WHO	World Health Organization
