# Supplementary material for: Intestinal Microbiome, Fecal Fermentation Profile, and Health Indices in HIV-Positive Men Versus Normal Controls Without HIV
Source: Nutrients. 2026 Jul 16;18(14):2328. doi: 10.3390/nu18142328 (PMC13415254; doi:10.3390/nu18142328)
Supplement: Supplementary file 1 [file nutrients-18-02328-s001.zip › nutrients-4377472-SI.pdf]

File S1: Informed Consent

Title of Research Study: *Evaluating differences in microbial fermentation profile, microbiome composition, dietary intake, CTRP3 protein concentration and liver profile of HAART HIV/AIDS and non-infected age matched individuals*
Principal Investigator: *William Andrew Clark, Ph.D.; phone: (423)-439-7708 and Jonathan Moorman, M.D.; phone: (423)-439-6380*
Organization of Principal Investigator: *East Tennessee State University*

INFORMED CONSENT

This Informed Consent will explain about being a participant in a research study. It is important that you read this material carefully and then decide if you wish to voluntarily participate.

A. **Purpose:** *The purpose(s) of this research study is/are as follows: We are evaluating differences in microbial fermentation profile, microbiome composition, dietary intake, CTRP3 protein concentration and liver profile of HAART HIV/AIDS and non-infected age matched individuals. We will collect fecal and blood samples from 20 participants with an HIV infection and undergoing treatment with HAART, and 20 non-infected participants to be used as a control base to see if there is a difference in expressed CTRP3 levels between infected and non-infected populations. You have been chosen as a candidate for this study because you have HIV, or you are a non-infected control candidate. We request that you tell us about any pre-existing medical conditions, medications, alcohol, or herbal supplements that you are currently taking. There will be no actual benefit to your current condition or illness as a result from this study.*

B. **Duration:** *The duration of this study will take as long as the study needs to recruit a total of twenty HAART treated HIV-infected individuals and matched (BMI and age) with 20 non-infected control participants. Your participation in the study only requires a short period of time (2-3 hours) that is needed to complete an informed consent, fill out a food frequency questionnaire, provide a complete fecal sample, receive a free liver scan to assess any fibrosis and/or percent fatty liver, and provide a blood sample taken by a trained technician.*

C. **Procedures:** *The procedures which will involve you as a research subject include: Research participants will complete an informed consent and food frequency questionnaire (Block 2014 Food Frequency Questionnaire). Subjects will provide a complete bowel movement sample (instructions and materials included), have their waist measured, receive a free-of-charge non-invasive liver scan to assess any fibrosis and/or percent fatty liver, and provide two tubes of blood (about 2 tablespoons) via venipuncture (a blood draw from an arm vein) by a trained technician after your permission. We will also obtain access to the HIV-infected subjects' medical records to obtain age, BMI, gender, and diagnoses. Blood samples and stool samples collected data will be used in this study. Non-infected subjects will only be required to self-report current medical condition/s to Andrew Clark, Ph.D. and submit their most current blood labs. No access to the "non-infected" subjects' medical record will be required. If you choose to withdraw from the study, all samples and medical records taken from the patient will be discarded according to safe and secure measures ensuring your privacy.*

D. **Alternative Procedures/Treatments:** *The alternative procedures/treatments available to you if you elect not to participate in this study are to continue with your routine care without participation. Participation or non-participation in this study will in no way alter your medical care.*

E. **Possible Risks/Discomforts:** *The possible risks and/or discomforts from your participation in this research study include the risks for blood draw which are no different than any other time you have*

had blood drawn. You may experience some discomfort from the needle puncture, bruising, or localized clot, but no risks are likely. Rarely, pain, bleeding from the puncture site, infection, or fainting may occur. Sterile technique will be used. Risks from providing a stool sample may include psychological shame/embarrassment as well as physical difficulty in collecting the sample. There is no potential risk or discomfort from the liver scan. Although unlikely, rarely there may be a possible risk of loss of confidentiality.

If you have any of the following, you cannot be in this study:

1. *If you are under the age of 18 or over the age of 60*
2. *HIV patients with viral load greater than 200*
3. *If you have Ulcerative Colitis*
4. *If you have Inflammatory Bowel Disease*
5. *If you have Crohn's Disease*

F. **Possible Benefits**: *If we see a difference in the colonic fermentation profile and microbiome that is different from the HIV/AIDS participants and the controls, we could envision a follow up study that would be an intervention with pre and probiotics. If we could change the leakiness of the gut we may be able to reduce the inflammation that the patient is seeing. There will be no actual benefit to your current condition or illness from this study.*

G. **Financial Costs**: *There are no additional costs to you as a result in participating in this study.*

H. **Compensation in the Form of Payments to Participant**: *You will receive compensation in the form of a twenty-dollar ($20.00) gift card for your participation in this research study. When blood and stool samples, questionnaire, and liver scan procedure have been collected, compensation in the form of a twenty-dollar ($20) gift card will be given to the participant on the same day of collection.*

I. **Voluntary Participation**: *Participation in this research experiment is voluntary. You may refuse to participate. You can quit at any time. If you quit or refuse to participate, the benefits or treatment to which you are entitled will not be affected. You may quit by calling Dr. William Andrew Clark, Ph.D. whose phone number is (423)-439-7708, and Dr. Jonathan Moorman, M.D., whose phone number is (423) 439-6380. You will be told immediately if any of the results of the study should reasonably be expected to make you change your mind about staying in the study. If you fail to submit a questionnaire, informed consent, fecal sample, or blood sample, or if you choose not to participate, then you will be removed from the study. Your Principal Investigator may take you out of the study at any time without your consent, if he decides it is not in your best interest to continue (i.e., not following study related directions, adverse event). You may be taken off the study if it ends early.*

J. **Body Fluids, Substances or Tissues**: *Fecal and blood samples will be processed on receipt. Fecal samples will be frozen for reanalysis by microbiome assay, fecal fermentation profile, gross energy, fatty acid profile, crude protein content, and fat percent. I authorize the use of my bodily fluids, substances and/or tissues for research purposes.*

K. Contact for Questions: *If you have any questions, problems or research-related medical problems at any time, you may call Dr. William Andrew Clark, Ph.D., whose phone number is*

(423)-439-7708, and Dr. Jonathan Moorman, M.D., whose phone number is (423) 439-6380. You may also call the Chairperson of the ETSU Institutional Review Board at (423)-439-6054 for any questions you may have about your rights as a research participant. If you have any questions or concerns about the research and want to talk to someone independent of the research team or you can't reach the study staff, you may call an IRB Coordinator at (423)-439-6055 or (423)-439-6002.

L. Confidentiality: *Every attempt will be made to see that your study results are kept confidential. The samples will be split between two locations. 50% of the samples will be kept in Lamb Hall in room number 337 at East Tennessee State University in a lockable -80 freezer. The other 50% of the samples will be located at James H. Quillen College of Medicine's Molecular Biology Core Facility located in Building 119 room 2-22 on the VA campus. The results of this study may be published and/or presented at meetings without naming you as a participant. Any data obtained will be stored on the encrypted ETSU server in Lamb Hall, room 338. The consent forms will be stored in a locking file cabinet in VA building 60, East Wing room 170. Although your rights and privacy will be maintained, the Secretary of the Department of Health and Human Services, the ETSU IRB, William Andrew Clark, PhD., Jonathan Moorman, M.D. and their research team have access to the study records. Your (medical) records will be kept completely confidential according to current legal requirements. They will not be revealed unless required by law, or as described in this form.*

HIPAA Authorization
Authorization for Disclosure of Protected Health Information for Research

M. Purpose: *The purpose of this authorization form is to authorize William Andrew Clark, Ph.D. ETSU, PO Box 70282, Johnson City, TN 37604, Jonathan Moorman, M.D., VA Building 3, Box 70622, Johnson City, TN 37604 and their research team to collect, use, and disclose your protected health information to conduct the research study listed above. This authorization will inform you what information about you may be collected in this study as well as who might see or use your information. East Tennessee State University has rules that require the research team to protect your health information. There are also federal and state laws that protect the privacy of your health information. Generally, only people on the research team will know that you are in the research study and will see your protected health information. However, there are a few exceptions that are listed in Section O of this form.*

By signing this authorization form, you authorize the research team to collect, use and disclose your health information as described in this form. ***You do not have to sign this form****. Your decision not to sign this authorization will not affect your treatment, healthcare, enrollment in health plans or eligibility for benefits. However, your decision not to sign this form will result in your not being allowed to participate in this research study.*

N. **Protected Health Information to be Used/Disclosed:** *Protected health information is the information in your medical or other healthcare records. This includes all information in your records that can identify you including your name, address, phone number, birth date, and account numbers.*

1. *By signing this form, you authorize the following healthcare providers, health plans, or other organizations or individuals to disclose your protected health information to the research team:*

   East Tennessee State University

   The research team will be collecting PHI from all healthcare providers in East Tennessee State University that have patients suitable for this study including Dr. Jonathan Moorman, MD.

2. *By signing this form, you authorize the individuals or organizations listed above to disclose the following types of protected health information to the research team:*
   - *Age*
   - *BMI*
   - *Gender*
   - *Diagnosis*
   - *Medical history/treatment notes*
   - *Hospital discharge summary*
   - *Radiology records*
   - *Radiology films*
   - *Laboratory/diagnostic tests*
   - *Pathology specimens and slides*
   - *Pathology reports*
   - *Diagnostic imaging reports*
   - *Results of physical exam*
   - *Immunologic assay results*
   - *Medical and laboratory records for healthcare rendered in connection with this research study, along with any other types of protected health information the research team will require.*

3. *By signing this form, you authorize the research team to collect, use and disclose your protected health information as listed above, in relation to health care provided to you as long as the study requires.*

O. **How your protected health information will be used:** *Dr. William Andrew Clark, Ph.D. and Dr. Jonathan Moorman, M.D. and their research team will collect, use and disclose the protected health information described in this form for the purpose of conducting the research study listed on this form. Generally, only Dr. William Andrew Clark, Ph.D. and Dr. Jonathan Moorman, M.D. and those individuals on the research team will see your protected health information. However, in*

certain circumstances the following individuals or organizations may have access to your protected health information:

1. *The Department of Health and Human Services*
2. *The ETSU Institutional Review Board*
3. *The ETSU Human Research Protection Program*
4. *The ETSU HIPAA Compliance Office*
5. *Other representatives of ETSU as reasonably required to carry out the research study*
6. *MEAC*
7. *Individuals at East Tennessee State University that are responsible for financial oversight of research including billing and payments*
8. *Other Individuals/Organizations as required by law*

P. Access to your Protected Health Information: *During the course of this research study, you will not be allowed to see or copy your protected health information created by Dr. William Andrew Clark, Ph.D. and Dr. Jonathan Moorman, M.D. in connection with this research study. You have the option for a debriefing at the end of the study regarding the research findings, their colonic fermentation profile, and/or their liver scan information. You will be allowed to see or copy these records at the conclusion of this study.*

Q. Redisclosure of your protected health information: *Once your protected health information is disclosed to anyone outside this research study, the information may no longer be protected by the federal privacy standards and may be redisclosed without obtaining your authorization Dr. William Andrew Clark, Ph.D. and Dr. Jonathan Moorman, M.D. and their research team will only collect, use and disclose your protected health information as described in this form or as otherwise permitted or required by law.*

R. Right to revoke this authorization: *If you sign this authorization form, you may change your mind at any time.* *If you change your mind, the research team may keep and use your protected health information that they already have. The research team will not obtain any more protected health information about you for this research unless permitted or required by law after you change your mind.*

In order to change your mind and revoke this authorization, you must send a written letter to: William Andrew Clark, Ph.D. ETSU, PO Box 70282, Johnson City, TN 37604, or Jonathan Moorman, M.D., VA Building 3, Box 70622, Johnson City, TN 37604

If you change your mind you will no longer be able to participate in this research study.

S. Expiration of authorization: *This authorization does not have an expiration date.*

T. **Questions about Privacy:** *If you have any questions or concerns about your privacy rights you may contact the East Tennessee State University HIPAA Compliance Office via telephone (423).439.8533 or mail P.O. Box 70285, Johnson City, TN 37614.*

By signing below, I confirm that I have read and understand both the Informed Consent and HIPAA Authorization sections of this form and that I had the opportunity to have them explained to me verbally. You will be given a signed copy of this informed consent document. I confirm that I have had the opportunity to ask questions and that all my questions have been answered. By signing below, I confirm that I freely and voluntarily choose to take part in this research study, and that I authorize Dr. William Andrew Clark, Ph.D. and Dr. Jonathan Moorman, M.D. and their research team to collect, use and disclose my protected health information as described in this form.

____
Signature of Subject *Date*

____
Printed Name of Subject *Date*

____
Signature of Person Obtaining Consent *Date*

You will be provided with a copy of this signed authorization form.

File S2: Block food frequency questionnaire.

FOOD AND ACTIVITY QUESTIONNAIRE

RESPONDENT ID #

0	0	0	0	0	0	0	0	0	0
1	1	1	1	1	1	1	1	1	1
2	2	2	2	2	2	2	2	2	2
3	3	3	3	3	3	3	3	3	3
4	4	4	4	4	4	4	4	4	4
5	5	5	5	5	5	5	5	5	5
6	6	6	6	6	6	6	6	6	6
7	7	7	7	7	7	7	7	7	7
8	8	8	8	8	8	8	8	8	8
9	9	9	9	9	9	9	9	9	9

TODAY'S DATE

Month	DAY		YEAR
○ Jan			
○ Feb			
○ Mar	0	0	2014 ○
○ Apr	1	1	2015 ○
○ May	2	2	2016 ○
○ Jun	3	3	2017 ○
○ Jul		4	2018 ○
○ Aug		5	2019 ○
○ Sep		6	2020 ○
○ Oct		7	2021 ○
○ Nov		8	2022 ○
○ Dec		9	2023 ○

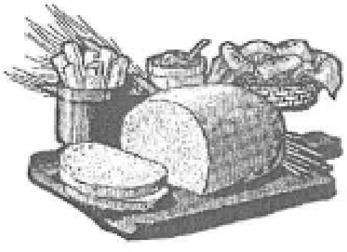

ABOUT YOU

SEX
○ Male
○ Female

If female, are you pregnant or breast feeding?
○ No
○ Yes
○ Not female

AGE | **WEIGHT** pounds | **HEIGHT** feet inches

AGE		WEIGHT			HEIGHT feet	HEIGHT inches
0	0	0	0	0		0
1	1	1	1	1		1
2	2	2	2	2		2
3	3	3	3	3	3	3
4	4	4	4	4	4	4
5	5		5	5	5	5
6	6		6	6	6	6
7	7		7	7		7
8	8		8	8		8
9	9		9	9		9
						10
						11

ABOUT THIS SURVEY

Please answer each question as best you can. Estimate if you aren't sure.

- **DETACH THE LAST PAGE OF THIS BOOKLET.** These are your portion pictures.
- **USE ONLY A NUMBER 2 PENCIL.**
- **FILL IN THE CIRCLES COMPLETELY** and erase completely if you make any changes.

© Copyright NutritionQuest © This form may not be used or reproduced without permission. Please call 510-704-8514 for reprints

INSTRUCTIONS

This form is about the foods you usually eat. Think about your usual intake over the last year. This includes all meals or snacks, at home, in a restaurant, or carry-out.

Please tell us...

1. **HOW OFTEN,** on average, did you eat the food?
   DO NOT SKIP any foods. Mark "Never" if you didn't eat any of the food.
2. **HOW MUCH** of the food did you usually eat on the days you ate it?
   Sometimes we ask "how much" as A, B, C or D. **LOOK AT THE PORTION PICTURES.**
   Pick the picture that looks the most like the serving size you usually eat.
   (If you don't have pictures: A=1/4 cup, B=1/2 cup, C=1 cup, D=2 cups.)
3. **WHAT TYPE?** For some foods we ask the type (low-fat, low-sugar...) near the end of the survey.

EXAMPLE: This person drank orange juice twice a week, and had one glass each time.
Once a week this person ate a "C"-sized serving of cold cereal (about 1 cup).

	HOW OFTEN in the past year?									HOW MUCH on those days? SEE PORTION SIZE PICTURES FOR A-B-C-D				
	NEVER	A FEW TIMES per YEAR	ONCE per MONTH	2-3 TIMES per MONTH	ONCE per WEEK	2 TIMES per WEEK	3-4 TIMES per WEEK	5-6 TIMES per WEEK	EVERY DAY					
Orange juice	○	○	○	○	○	●	○	○	○	How many glasses	● 1	○ 2	○ 3	○ 4
Cold cereal	○	○	○	○	●	○	○	○	○	Which bowl	○ A	○ B	● C	○ D

PLEASE DO NOT WRITE IN THIS AREA

SERIAL #

EGGS and DAIRY FOODS	NEVER	A FEW TIMES per YEAR	ONCE per MONTH	2-3 TIMES per MONTH	ONCE per WEEK	2 TIMES per WEEK	3-4 TIMES per WEEK	5-6 TIMES per WEEK	EVERY DAY	HOW MUCH on those days? SEE PORTION SIZE PICTURES FOR A-B-C-D				
Breakfast sandwiches or breakfast burritos with eggs or meat	○	○	○	○	○	○	○	○	○	How many sandwiches in a day	○ 1	○ 2		
Other eggs like scrambled or boiled, or quiche (not egg substitutes)	○	○	○	○	○	○	○	○	○	How many eggs a day	○ 1	○ 2	○ 3	○ 4
Yogurt (not frozen yogurt)	○	○	○	○	○	○	○	○	○	Which bowl		○ B	○ C	○ D
Cottage cheese, ricotta cheese	○	○	○	○	○	○	○	○	○	How much	○ A	○ B	○ C	○ D
Cream cheese, sour cream, dips	○	○	○	○	○	○	○	○	○	How many tablespoons	○ 1	○ 2	○ 3	○ 4
Cheese, sliced cheese, cheese spread, including in sandwiches and quesadillas	○	○	○	○	○	○	○	○	○	How many slices	○ 1	○ 2	○ 3	○ 4
**CEREALS, GRAINS, BREADS**														
Cold cereals, ANY KIND, like corn flakes, fiber cereals, sweetened cereals	○	○	○	○	○	○	○	○	○	Which bowl		○ B	○ C	○ D
Oatmeal, or whole grain cereal like Wheatena or Ralston	○	○	○	○	○	○	○	○	○	Which bowl	○ A	○ B	○ C	○ D
Grits, cream of wheat, cornmeal mush	○	○	○	○	○	○	○	○	○	Which bowl	○ A	○ B	○ C	○ D
Milk or milk substitutes on cereal	○	○	○	○	○	○	○	○	○					
Brown rice, or dishes made with brown rice	○	○	○	○	○	○	○	○	○	How much in a day		○ B	○ C	○ D
White rice, or dishes made with white rice, like rice and beans	○	○	○	○	○	○	○	○	○	How much in a day		○ B	○ C	○ D
Pancakes, waffles, French toast, crepes	○	○	○	○	○	○	○	○	○	How many	○ 1	○ 2	○ 3	○ 4
Breakfast pastries, like muffins, scones, sweet rolls, Danish, Pop Tarts, pan dulce	○	○	○	○	○	○	○	○	○	How many pieces	○ 1 sm	○ 1 med	○ 2	○ 3
Biscuits, not counting breakfast sandwiches	○	○	○	○	○	○	○	○	○	How many	○ 1 sm	○ 1 med	○ 2	○ 3
Corn bread, corn muffins, hush puppies	○	○	○	○	○	○	○	○	○	How many pieces in a day	○ 1/2	○ 1	○ 2	○ 3
Hamburger buns, hotdog buns, submarine or hoagie buns	○	○	○	○	○	○	○	○	○	How many buns in a day	○ 1/2	○ 1	○ 2	○ 3
Bagels or English muffins, dinner rolls, pita, naan	○	○	○	○	○	○	○	○	○	How many	○ 1/2	○ 1	○ 2	○ 3
Tortillas (not counting in tacos or burritos)	○	○	○	○	○	○	○	○	○	How many in a day	○ 1	○ 2	○ 3	○ 4
Any other bread or toast, including white, dark, whole wheat, and what you have in sandwiches	○	○	○	○	○	○	○	○	○	How many slices in a day	○ 1	○ 2	○ 3	○ 4
**VEGETABLES**														
Broccoli, Chinese broccoli, or Brussels sprouts	○	○	○	○	○	○	○	○	○	How much	○ A	○ B	○ C	○ D
Carrots and mixed vegetables containing carrots	○	○	○	○	○	○	○	○	○	How much	○ A	○ B	○ C	○ D
Corn	○	○	○	○	○	○	○	○	○	How much	○ A	○ B	○ C	○ D
Green beans, string beans, green peas	○	○	○	○	○	○	○	○	○	How much	○ A	○ B	○ C	○ D
Cooked greens like spinach, collards, turnip greens, kale, mustard greens	○	○	○	○	○	○	○	○	○	How much	○ A	○ B	○ C	○ D

© Copyright NutritionQuest
This form may not be used or reproduced without permission
Please call 510-[illegible] for reprints

	NEVER	A FEW TIMES per YEAR	ONCE per MONTH	2-3 TIMES per MONTH	ONCE per WEEK	2 TIMES per WEEK	3-4 TIMES per WEEK	5-6 TIMES per WEEK	EVERY DAY	HOW MUCH on those days? SEE PORTION SIZE PICTURES FOR A-B-C-D				
Cabbage, cole slaw, Chinese cabbage										How much	A	B	C	D
Green salad with lettuce or raw spinach										How much	1/2 cup	1 cup	2 cups	3+ cups
Raw tomatoes										How much	1/4	1/2	1	2
Salad dressing										How many tablespoons	1	2	3	4
Avocado, guacamole										How many tablespoons	1	2	3	4
Sweet potatoes, yams										How much	A	B	C	D
French fries, home fries, hash browns, tater tots										How much	A	B	C	D
Potatoes not fried, like baked, boiled, mashed, or in stew or potato salad										How much	A	B	C	D
Any other vegetable, like squash, cauliflower, peppers, okra, nopales										How much	A	B	C	D
**FRUITS**														
**How often do you eat the following 2 items, just during the summer months when they are in season?**														
Watermelon, cantaloupe, honeydew, other melons, in season										How much	A	B	C	D
Strawberries or other berries, in season										How much	A	B	C	D
**How often do you eat the following fruits all year round? Estimate your average for the whole year. Include fresh or frozen fruits. Only include canned or dried fruit when mentioned.**														
Bananas										How many in a day	1/2	1	2	
Apples or pears										How many in a day	1/2	1	2	
Oranges, tangerines, grapefruit										How many	1/2	1	2	
Peaches and nectarines										How many	1/2	1	2	
Any other fresh fruit, like grapes, plums, mango, fruit salad										How much	A	B	C	D
Raisins, dates, other dried fruit										How much	A	B	C	
Canned fruit, like applesauce, fruit cocktail, canned peaches or pineapple										How much	A	B	C	D
**BEANS, TOFU, and MEAT SUBSTITUTES** Include those eaten alone, or in mixed dishes like burritos, chili, stir-fry, salad														
Refried beans, bean burritos, or hummus										How much	A	B	C	D
Pinto beans, black beans, kidney beans, baked beans, lentils										How much	A	B	C	D
Tofu or tempeh										How much	A	B	C	D
Meat substitutes, like veggie burgers, veggie chicken, vegetarian hot dogs or vegetarian lunch meats										How much	A	B 1 patty or slice	C	D

PLEASE DO NOT WRITE IN THIS AREA

SERIAL #

SOUPS, MIXED DISHES, and NOODLES	NEVER	A FEW TIMES per YEAR	ONCE per MONTH	2-3 TIMES per MONTH	ONCE per WEEK	2 TIMES per WEEK	3-4 TIMES per WEEK	5-6 TIMES per WEEK	EVERY DAY	HOW MUCH on those days? SEE PORTION SIZE PICTURES FOR A-B-C-D	
Split pea, bean, or lentil soup										Which bowl	B C D
Vegetable soup, vegetable beef soup, or tomato soup										Which bowl	B C D
Any other soup, including chicken noodle, cream soups, Cup-A-Soup, ramen										Which bowl	B C D
Pizza or pizza pockets										How many slices	1 2 3 4
Macaroni and cheese										How much	B C D
Spaghetti, lasagna, other pasta with tomato sauce										How much	B C D
Other noodles like plain pasta, pasta salad, sopa seca										How much	B C D
Egg rolls, won tons, samosas, empanadas										How many pieces	1 2 3 4
**MEAT and CHICKEN**											
Hamburgers, cheeseburgers, turkey burger, at home or from a restaurant										How many	1 sm, 1 lrg, 2, 3
Hot dogs or dinner sausage like Polish, Italian, chicken apple										How many	1 2 3 4
Bacon or breakfast sausage										How many pieces	1 2 3 4
Lunch meats like bologna, sliced ham, sliced turkey, salami										How many slices	1 2 3 4
Meat loaf, meat balls										How much	B C D
Steak, roast beef, pot roast, including in frozen dinners or sandwiches										How much	A B C D
Tacos, burritos, enchiladas, tamales, tostadas, with meat or chicken										How much	A B C D
Ribs, spareribs										How much	A B C D
Pork chops, pork roast, cooked ham (including for breakfast)										How much	A B C D
Any other beef or pork dish like stew, pot pie, corned beef hash, chili, Hamburger Helper, curry										How much	B C D
Liver, including chicken livers or liverwurst										How much	A B C
Pigs feet, neck bones, oxtails, tongue, chitlins										How much	A B C
Veal, lamb, goat, deer meat, other game										How much	A B C
Fried chicken, including chicken fingers, chicken nuggets, wings, chicken patty										How many medium pieces	1, 2 pcs/6 nuggets, 3, 4
Roasted or broiled chicken or turkey										How much	A, B medium piece, C, D half chicken
Any other chicken or turkey dish, like chicken stew or curry, chicken salad, stir-fry, Chinese chicken dishes										How much	B C D

This form may not be used or reproduced without permission. © Copyright NutritionQuest. Please call 510-704-8514 for reprints

FISH, SEAFOOD	NEVER	A FEW TIMES per YEAR	ONCE per MONTH	2-3 TIMES per MONTH	ONCE per WEEK	2 TIMES per WEEK	3-4 TIMES per WEEK	5-6 TIMES per WEEK	EVERY DAY	HOW MUCH on those days? SEE PORTION SIZE PICTURES FOR A-B-C-D				
Oysters										How much	A	B	C	
Shellfish like shrimp, scallops, crab										How much fish	A	B	C	D
Tuna, tuna salad, tuna casserole										How much of the tuna	A	B	C	
Salmon, mackerel, sea bass, trout, sardines, herring, <u>without breading</u>										How much	A	B	C	D
Fried fish, fish sticks, fish sandwich, <u>breaded</u> fillets										How much	A	B	C	D
Any other fish										How much fish	A	B	C	D

NUTS, SEEDS, SNACKS	NEVER	A FEW TIMES per YEAR	ONCE per MONTH	2-3 TIMES per MONTH	ONCE per WEEK	2 TIMES per WEEK	3-4 TIMES per WEEK	5-6 TIMES per WEEK	EVERY DAY	HOW MUCH on those days?				
Peanut butter or other nut butters										How many tablespoons	1/2	1	2	3
Walnuts or flax seeds (don't count flaxseed oil)										How much	1 Tbsp	2 Tbsp	1/4 cup	1/2 cup
Peanuts, sunflower seeds, other nuts or seeds										How much	A	B	C	D
Energy or protein bars, like Power Bar, Clif, Balance, Luna, South Beach, Atkins										How much	Small	Medium	Large	
Breakfast bars, cereal bars, granola bars (<u>not</u> energy or protein bars)										How many	1	2	3	
Popcorn										How many cups	1-2	3-6	7-9	10-12
<u>Whole grain</u> crackers, like Wheat Thins, RyeKrisp, Ryvita, Wasa										How much	A	B	C	D
Any other crackers, like saltines, Ritz, Cheez-Its, cheese-filled pretzels										How much	A	B	C	D
Tortilla chips or corn chips, like Fritos, Doritos, corn nuts										How much	A	B	C	D
Any other snack chips, like potato chips, Cheetos, Chex mix										How much	A	B	C	D

SWEETS AND DESSERTS	NEVER	A FEW TIMES per YEAR	ONCE per MONTH	2-3 TIMES per MONTH	ONCE per WEEK	2 TIMES per WEEK	3-4 TIMES per WEEK	5-6 TIMES per WEEK	EVERY DAY	HOW MUCH on those days?				
Donuts										How many	1 mini	1 med	2	3
Cake or snack cakes like cupcakes, Twinkies, pound cake, banana bread										How many pieces	1 sm	1 med	2	3
Cookies, brownies										How many	1-2	3-4	5-6	7+
Pumpkin pie, sweet potato pie										How many pieces	1/2	1	2	3
Any other pie or cobbler, including fast food pies, snack pies										How many pieces	1/2	1	2	3
Ice cream, ice cream bars, frozen yogurt, fast food milkshakes										How much		B	C	D
Pudding, custard, rice pudding, flan										How much		B	C	D
Chocolate or other flavored sauces or syrup, on ice cream										How much	1-2 Tbspn	3-4 Tbspn	1/2 cup	

© Copyright NutritionQuest
This form may not be reproduced without written permission
Please call 510-704-8514 for reprints

PLEASE DO NOT WRITE IN THIS AREA

SERIAL #

	NEVER	A FEW TIMES per YEAR	ONCE per MONTH	2-3 TIMES per MONTH	ONCE per WEEK	2 TIMES per WEEK	3-4 TIMES per WEEK	5-6 TIMES per WEEK	EVERY DAY	HOW MUCH on those days? SEE PORTION SIZE PICTURES FOR A-B-C-D
Popsicles, jello, frozen fruit bars, slushies, sherbet (don't count sugar-free)										How much: A / B / C / D
Chocolate candy, candy bars like Snickers, Hershey's, M&Ms										How much in a day: 1 mini / 1 med / 1 lrg / 1 king
Any other candy, not chocolate, like hard candy, Lifesavers, Skittles, Starburst, breath mints, chewing gum (NOT sugar free)										How much in a day: 1-2 pcs / 1/2 pkg / 1 pkg / 2 pkgs
**SPREADS, SAUCES, OTHER FOODS**										
Margarine (not butter) on bread, rice, vegetables, or other foods										How many pats (tsps): 1 / 2 / 3 / 4
Butter (not margarine) on bread, rice, vegetables, or other foods										How many pats (tsps): 1 / 2 / 3 / 4
Mayonnaise, sandwich spreads										How many tablespoons: 1/2 / 1 / 2 / 3
Ketchup, salsa, chili sauce, chili peppers										How many tablespoons: 1/2 / 1 / 2 / 3
Mustard, barbecue sauce, soy sauce										How many tablespoons: 1/2 / 1 / 2 / 3
Gravy, or other rich sauces like Alfredo, white sauce, mole, peanut sauce										How many cups: 1/4 / 1/2 / 1
Jam, jelly, marmalade										How many tablespoons: 1/2 / 1 / 2 / 3
Pickles, pickled vegetables, sauerkraut, kimchi										How much: A / B / C / D
Salt, added to your food at the table										How many shakes in a day: 1-3 / 4-5 / 6-7 / 8+
**BEVERAGES**										
Chocolate milk, cocoa, hot chocolate										How many 12 ounce servings: 1/2 / 1 / 2 / 3
Glasses of milk or soy milk, (not counting on cereal, in coffee, or chocolate milk)										How many 8 ounce servings: 1 / 2 / 3 / 4
Meal replacement drinks like Slim Fast, Ensure, or high protein drinks or powders										How many cans or glasses: 1 / 2 / 3 / 4
Tomato juice, V-8, other vegetable juice										How many 8 ounce servings: 1/2 / 1 / 2 / 3
Real 100% orange juice or grapefruit juice. Don't count orange soda or Sunny Delight.										How many 8 ounce servings: 1/2 / 1 / 2 / 3
Other 100% juices, like apple, grape, 100% fruit blends, or fruit smoothies										How many 8 ounce servings: 1/2 / 1 / 2 / 3
Hi-C, cranberry juice cocktail, Hawaiian Punch, Tang										How many 12 ounce servings: 1/2 / 1 / 2 / 3
Drinks with some juice like Sunny Delight, Knudsen										How many 12 ounce servings: 1/2 / 1 / 2 / 3
Iced tea, homemade, instant or bottled, like Nestea, Lipton, Snapple, Tazo										How many 16-oz. glasses or bottles: 1/2 / 1 / 2 / 3
Gatorade, Powerade, or other sports drinks										How much in a day: 1 16-ounce bottle / 1 20-ounce bottle / 2 16-ounce bottles / 2 20-ounce bottles

© Copyright NutritionQuest. This form may not be used or reproduced without permission. Please call 510-704-8514 for reprints.

	NEVER	A FEW TIMES per YEAR	ONCE per MONTH	2-3 TIMES per MONTH	ONCE per WEEK	2 TIMES per WEEK	3-4 TIMES per WEEK	5-6 TIMES per WEEK	EVERY DAY	HOW MUCH on those days? SEE PORTION SIZE PICTURES FOR A-B-C-D
Energy drinks like Red Bull, Rockstar, Monster										How much in a day: 1 8-ounce can / 1 12-16 ounce can / 1 20-ounce can / 24 ounces or more
Kool-Aid, lemonade, fruit flavored drinks, like Crystal Light, atole, horchata (<u>not</u> iced tea)										How much in a day: 1 8-ounce glass / 1 12-16-ounce glass or bottle / 1 20-ounce bottle / 30 ounces or more
Soft drinks, soda, pop, like cola, 7-Up, orange soda, regular or diet										How many in a day: 1 can / 1 20-ounce bottle / 2 cans / Big Gulp or 3 cans
Beer or non-alcoholic beer										How much in a day: 1 can / 2 cans / 3-4 cans or small pitcher / 5+ cans or large pitcher
Wine or wine coolers										How many glasses in a day: 1/2 glass / 1 glass / 2 glasses, 1/2 bottle / 4+ glasses
Liquor or mixed drinks, cocktails										How many drinks: 1 / 2 / 3 / 4
Water, bottled or tap										How many glasses: 1 / 2 / 3-4 / 5+
Milky coffee drinks like latte, mocha, cappuccino, Frappuccino										How much in a day: 12 oz / 16 oz / 20 oz / 24+ oz
Coffee (brewed or instant), regular or decaf										How many in a day: 1 / 2 / 3 / 4+
Hot tea (<u>not</u> including herbal tea)										How many cups in a day: 1 / 2 / 3 / 4+

<u>MILKY COFFEE DRINKS:</u> What kind do you usually drink? MARK ONLY ONE

◯ Frappuccino ◯ Mocha ◯ Latte or cappuccino ◯ Café con leche ◯ Some of each ◯ Don't drink them

What are your milky coffee drinks usually made with? **MARK ONLY ONE**

◯ Whole milk ◯ Skim milk or non-fat ◯ Something else
◯ 1 or 2% milk (reduced fat) ◯ Soy milk ◯ Don't drink

<u>COFFEE:</u> Is your coffee usually regular or decaf? ◯ Decaf ◯ Regular ◯ Both kinds ◯ Don't drink coffee

What do you usually add to your regular or decaf coffee? **MARK ONLY ONE**

◯ Cream or half-n-half ◯ Condensed milk ◯ None of these
◯ CoffeeMate, non-dairy creamer ◯ Any other milk

Do you usually add sugar (or honey) to coffee? ◯ No ◯ Yes IF YES, how many teaspoons <u>each cup</u>? ◯ 1 ◯ 2 ◯ 3 ◯ 4

<u>HOT TEA:</u> Is your hot tea usually regular or decaf? ◯ Decaf ◯ Regular ◯ I drink both kinds ◯ Don't drink tea

What do you usually add to your hot tea? **MARK ONLY ONE**

◯ Cream or half-n-half ◯ Condensed milk ◯ None of these
◯ CoffeeMate, non-dairy creamer ◯ Any other milk

Do you usually add sugar (or honey) to hot tea? ◯ No ◯ Yes IF YES, how many teaspoons <u>each cup</u>? ◯ 1 ◯ 2 ◯ 3 ◯ 4

This form may not be used or reproduced without permission. © Copyright NutritionQuest ®
Please call 510-704-8554 for reprints

If you eat the following foods, what type do you usually eat? MARK ONLY ONE ANSWER FOR EACH QUESTION

Food	Answer options
**Milk**	○ Whole milk ○ 2% milk ○ 1% milk (low-fat) ○ Skim milk, non-fat ○ Soy milk ○ Rice milk ○ Almond milk, other ○ Don't drink
**Slimfast, Ensure, or high protein drinks**	○ Slimfast, Ensure, regular ○ Slimfast, Ensure, light or low-carb ○ High protein drinks, regular ○ High protein drinks, light or low-carb ○ Don't know/Don't drink
**Real 100% orange or grapefruit juice**	○ Calcium-fortified ○ Not calcium-fortified ○ Don't know ○ Don't drink
**Iced tea**	○ Home-made, no sugar ○ Bottled, no-sugar ○ Don't drink ○ Home-made, with sugar ○ Bottled, pre-sweetened
**Drinks like Kool-Aid, lemonade, Crystal Light**	○ Low-calorie, sugar-free ○ Regular ○ Don't drink
**Energy drinks like Red Bull, Monster**	○ Sugar-free ○ Regular ○ Don't drink
**Soft drinks, soda, pop**	○ Diet, low-calorie ○ Regular ○ Don't drink
**Do they usually have caffeine?**	○ Has caffeine ○ No caffeine ○ Don't drink
**Beer**	○ Regular ○ Light ○ Non-alcoholic ○ Don't drink
**Wine or wine cooler**	○ Red wine ○ White wine ○ Both red and white wine ○ Don't drink
**Cheese**	○ Low-fat ○ Regular-fat ○ Don't eat
**Yogurt**	○ Plain (no sugar or fruit) ○ With fruit or other flavors
**Yogurt**	○ Low-fat ○ Non-fat ○ Regular (whole milk) ○ Don't eat
**Salad dressing**	○ Low-fat, lite ○ Fat free ○ Regular ○ Oil & vinegar ○ Don't use
**Spaghetti or lasagna**	○ Meatless ○ With meat sauce or meatballs ○ Don't eat
**Noodles, pasta**	○ Rarely whole grain ○ Sometimes whole grain ○ Usually whole grain ○ Don't know/don't eat
**Burgers**	○ Hamburger ○ Cheeseburger ○ Turkey burger ○ Don't eat
**Beef or pork**	○ Avoid eating the fat ○ Sometimes eat the fat ○ Often eat the fat ○ Don't eat
**Chicken or turkey**	○ Avoid eating the skin ○ Sometimes eat the skin ○ Often eat the skin ○ Don't eat
**Hot dogs, dinner sausage**	○ Beef or pork ○ Chicken or turkey, low-fat ○ Don't eat
**Lunch meats**	○ Beef or pork ○ Chicken or turkey, low-fat ○ Don't eat
**Cakes, snack cakes, cupcakes**	○ Low-sugar, low-carb ○ Low-fat ○ Regular-fat ○ Don't eat
**Cookies, brownies**	○ Low-sugar, low-carb ○ Low-fat ○ Regular-fat ○ Don't eat
**Ice cream, frozen yogurt**	○ Low-sugar, low-carb ○ Low-fat or frozen yogurt ○ Regular ○ Don't eat
**Energy or protein bars**	○ High energy ○ High protein ○ Some of each ○ Don't know ○ Don't eat
**Bagels, English muffins, rolls**	○ White ○ Multi-grain ○ 100% whole wheat ○ Eat all kinds ○ Don't eat
**Burger, hot dog, submarine buns**	○ White ○ Multi-grain ○ 100% whole wheat ○ Eat all kinds ○ Don't eat
**Bread**	○ White (not whole grain) ○ 100% whole wheat ○ Don't eat ○ Multi-grain, rye, or other brown bread ○ Eat some of each
**Tortillas**	○ Corn tortillas ○ Flour tortillas (wheat) ○ Eat all kinds or don't know ○ Don't eat
**Popcorn**	○ Air popped, fat-free ○ Low-fat or light ○ Regular ○ Caramel corn ○ Don't know ○ Don't eat
**Crackers, pretzels**	○ Low-fat, including RyeKrisp, rice cakes, or plain pretzels ○ Don't know ○ Regular-fat crackers or filled pretzels ○ Don't eat
**Mayonnaise or sandwich spreads**	○ Low-fat, light ○ Regular ○ Don't eat

This form [illegible] © Copyright [illegible] for reprints

If you eat **cold cereals**, what do you usually eat? Choose **ONE or TWO** that you eat most often. If you usually eat just one kind, only choose one.

○ All-Bran Original	○ Cinnamon Toast Crunch	○ Grape Nuts	○ Special K, plain
○ All-Bran Complete, Complete	○ Cocoa Krispies, Pebbles, Puffs	○ Honey Bunches of Oats	○ Special K, flavors
○ Apple Jacks, Cookie Crisp	○ Corn Flakes, Corn Puffs	○ Kashi GOLEAN, Heart to Heart	○ Total
○ Bran Flakes	○ Corn Pops	○ Life	○ Wheaties
○ Cap'n Crunch	○ Fiber-One, Bran Buds	○ Lucky Charms, Fruity Pebbles	○ *Other sweet cereal*
○ Cheerios, plain or Multi-Grain	○ Froot Loops	○ Oatmeal Squares, Oat Bran	○ *Other unsweetened cereal*
○ Cheerios, Honey Nut, flavors	○ Frosted Flakes	○ Raisin Bran	○ *Other whole grain cereal*
○ Chex, Wheat	○ Frosted Mini-Wheats	○ Rice Krispies, puffed rice	○ *Other bran or fiber cereal*
○ Chex, other	○ Granola	○ Shredded Wheat	○ *Don't eat cereal*

Which **fats or oils** are used most often for **cooking or frying** (not baking) in your home? **MARK ONLY ONE OR TWO**

○ Non-stick spray or none	○ Soft tub margarine	○ Corn oil, vegetable oil and blends	○ Other oil
○ Butter or ghee	○ Low-fat margarine	○ Peanut oil	○ Don't know
○ Butter/margarine blend	○ Olive oil	○ Lard, fatback, or bacon fat	
○ Stick margarine	○ Canola oil, safflower oil	○ Vegetable shortening, Crisco	

PLEASE DO NOT WRITE IN THIS AREA

SERIAL #

What vitamin supplements do you take fairly regularly?

Multiple Vitamins. Do you take...	HOW OFTEN: DIDN'T TAKE	A FEW DAYS per MONTH	1 DAY per WEEK	2 DAYS per WEEK	3-4 DAYS per WEEK	5-6 DAYS per WEEK	EVERY DAY	FOR HOW MANY YEARS?: LESS THAN 1 YEAR	1-4 YEARS	5-9 YEARS	10+ YEARS
Prenatal vitamins	○	○	○	○	○	○	○	○	○	○	○
Regular One-A-Day, Centrum, "senior" vitamins or house brands of multiple vitamins	○	○	○	○	○	○	○	○	○	○	○
Stress-tabs or B-Complex type	○	○	○	○	○	○	○	○	○	○	○
Antioxidant combination, eye formula	○	○	○	○	○	○	○	○	○	○	○
**Single Vitamins or Minerals,** taken alone or in combination. Do not count what is in your multiple vitamins above.											
Vitamin A (not beta-carotene)	○	○	○	○	○	○	○	○	○	○	○
Vitamin B-6	○	○	○	○	○	○	○	○	○	○	○
Vitamin B-12	○	○	○	○	○	○	○	○	○	○	○
Vitamin C	○	○	○	○	○	○	○	○	○	○	○
Vitamin D	○	○	○	○	○	○	○	○	○	○	○
Vitamin E	○	○	○	○	○	○	○	○	○	○	○
Folic acid, folate	○	○	○	○	○	○	○	○	○	○	○
Calcium or antacids with calcium, like Tums	○	○	○	○	○	○	○	○	○	○	○
Iron	○	○	○	○	○	○	○	○	○	○	○
Zinc	○	○	○	○	○	○	○	○	○	○	○
Cod liver oil, other fish oils, omega-3, flax seed oil, algae	○	○	○	○	○	○	○	○	○	○	○
Fiber supplements like Benefiber, Metamucil	○	○	○	○	○	○	○	○	○	○	○

© Copyright NutritionQuest. This form may not be used or reproduced without permission. Please call 510-704-8514 for reprints

If you take One-A-Day, Centrum or other types of multiple vitamins, do you usually take types that

○ Contain minerals, iron, zinc, etc. ○ Do not contain minerals ○ Don't know

If you take vitamin C, how many milligrams of **vitamin C** do you usually take, on the days you take it? (Select the closest amount)

○ 100 ○ 250 ○ 500 ○ 750 ○ 1000 ○ 1500 ○ 2000 ○ 3000+ ○ Don't know

If you take vitamin E, how many IUs of **vitamin E** do you usually take, on the days you take it? (Select the closest amount)

○ 100 ○ 200 ○ 300 ○ 400 ○ 600 ○ 800 ○ 1000 ○ 2000+ ○ Don't know

If you take calcium, how many milligrams of **calcium** do you usually take, on the days you take it? (Select the closest amount)

○ 100 ○ 350 ○ 650 ○ 1250+ ○ Don't know

If you take vitamin D, how many IUs of **vitamin D** do you usually take, on the days you take it? (Select the closest amount)

○ 400 ○ 600 ○ 800 ○ 1000 ○ 2000 ○ 3000 ○ 4000 ○ 5000+ ○ Don't know

If you take omega-3 supplements, what type do you usually take? **MARK ALL THAT APPLY**

○ Fish oil ○ Flax oil, hemp oil, other seed oil ○ Krill oil ○ Algae ○ Don't know

SOME LAST QUESTIONS ABOUT YOU

	RARELY	1-2 per WEEK	3-4 per WEEK	5-6 per WEEK	1 per DAY	1 1/2 per DAY	2 per DAY	3 per DAY	4+ per DAY
About how many servings of vegetables do you eat, not counting salad or potatoes? 1 serving = 1/2 cup.									
About how many servings of fruit do you eat, not counting juices? 1 serving = 1/2 cup or 1 medium fruit.									
How often do you eat foods prepared at home that are <u>cooked or fried</u> in **fat or oil?**									

During a regular day, how many **meals and snacks** do you usually eat?

Meals per day ○ 1 ○ 2 ○ 3 ○ 4 ○ 5+

Snacks per day ○ 1 ○ 2 ○ 3 ○ 4 ○ 5+

PHYSICAL ACTIVITY SURVEY

Think about the last 12 months. How often did you do the activities listed below?

	HOW OFTEN IN THE PAST YEAR					
	RARELY OR NEVER	A FEW TIMES A MONTH	ONCE OR TWICE A WEEK	3-4 TIMES A WEEK	5-6 TIMES A WEEK	ALMOST EVERY DAY
Cooking, shopping, light cleaning like doing laundry or dusting, or running errands						
Slow walking like walking the dog, or <u>light</u> work around the house like watering						
Work on the job involving standing, like store clerk, or work involving driving (like truck driver)						
Taking care of children (feeding, dressing), or moderate housework like sweeping, mopping, cleaning the tub						
Weeding, raking, mowing the lawn, or light house repairs						
Brisk walking, dancing, hunting or fishing, golf (NOT using a golf cart), or 'friendly' outdoor games like softball						
Factory work, mechanic, restaurant work, or work involving walking, like mail carrier						
Construction, painting, feeding livestock, or homecare like caring for an adult family member						
Heavy work like moving boxes, heavy digging or shoveling snow, farm chores like baling hay, or other HARD labor						
Exercising at the gym or at home, aerobics, weight training, jogging, or vigorous sports like basketball, soccer, tennis						
Bicycling or swimming for exercise						

	HOW MUCH TIME ON THOSE DAYS			
	LESS THAN 30 MINUTES	30-60 MINUTES	1-2 HOURS	3 OR MORE HOURS
Cooking, shopping, light cleaning like doing laundry or dusting, or running errands				
Slow walking like walking the dog, or <u>light</u> work around the house like watering				
Work on the job involving standing, like store clerk, or work involving driving (like truck driver)				
Taking care of children (feeding, dressing), or moderate housework like sweeping, mopping, cleaning the tub				
Weeding, raking, mowing the lawn, or light house repairs				
Brisk walking, dancing, hunting or fishing, golf (NOT using a golf cart), or 'friendly' outdoor games like softball				
Factory work, mechanic, restaurant work, or work involving walking, like mail carrier				
Construction, painting, feeding livestock, or homecare like caring for an adult family member				
Heavy work like moving boxes, heavy digging or shoveling snow, farm chores like baling hay, or other HARD labor				
Exercising at the gym or at home, aerobics, weight training, jogging, or vigorous sports like basketball, soccer, tennis				
Bicycling or swimming for exercise				

[illegible] Nutrition[illegible] may not be used or reproduced without permission [illegible] call 510-704-8514 for reprints

Are you ○ Hispanic or Latino ○ Not Hispanic or Latino ○ Do not wish to provide this information

What race do you consider yourself to be? **MARK ALL THAT APPLY**

○ White ○ Asian ○ Native Hawaiian or Other Pacific Islander

○ Black or African American ○ American Indian or Alaska Native ○ Do not wish to provide this information

Thank you very much for filling out this questionnaire.

Please take a minute to go back and fill in anything you may have skipped.

PLEASE DO NOT WRITE IN THIS AREA

SERIAL #

1/4" SPINE PERF

Fold along this line, then carefully detach.

Fold this page along the dotted line, then CAREFULLY detach this page. Your portion pictures are on the back.

© Copyright NutritionQuest ©
This form may not be used or reproduced without permission.
Please call 510-704-8514 for reprints

© www.NutritionQuest.com, CA (510) 704-8514.

Fold along this line, then carefully detach.

Portion Size Choices

Keep this in front of you while you are filling out The Food Questionnaire. You may use either the plates or the bowls to help you choose your usual portion size.

Choose A, B, C or D: A = 1/4 Cup of Food **B** = 1/2 Cup of Food C = 1 Cup of Food **D** = 2 Cups of Food

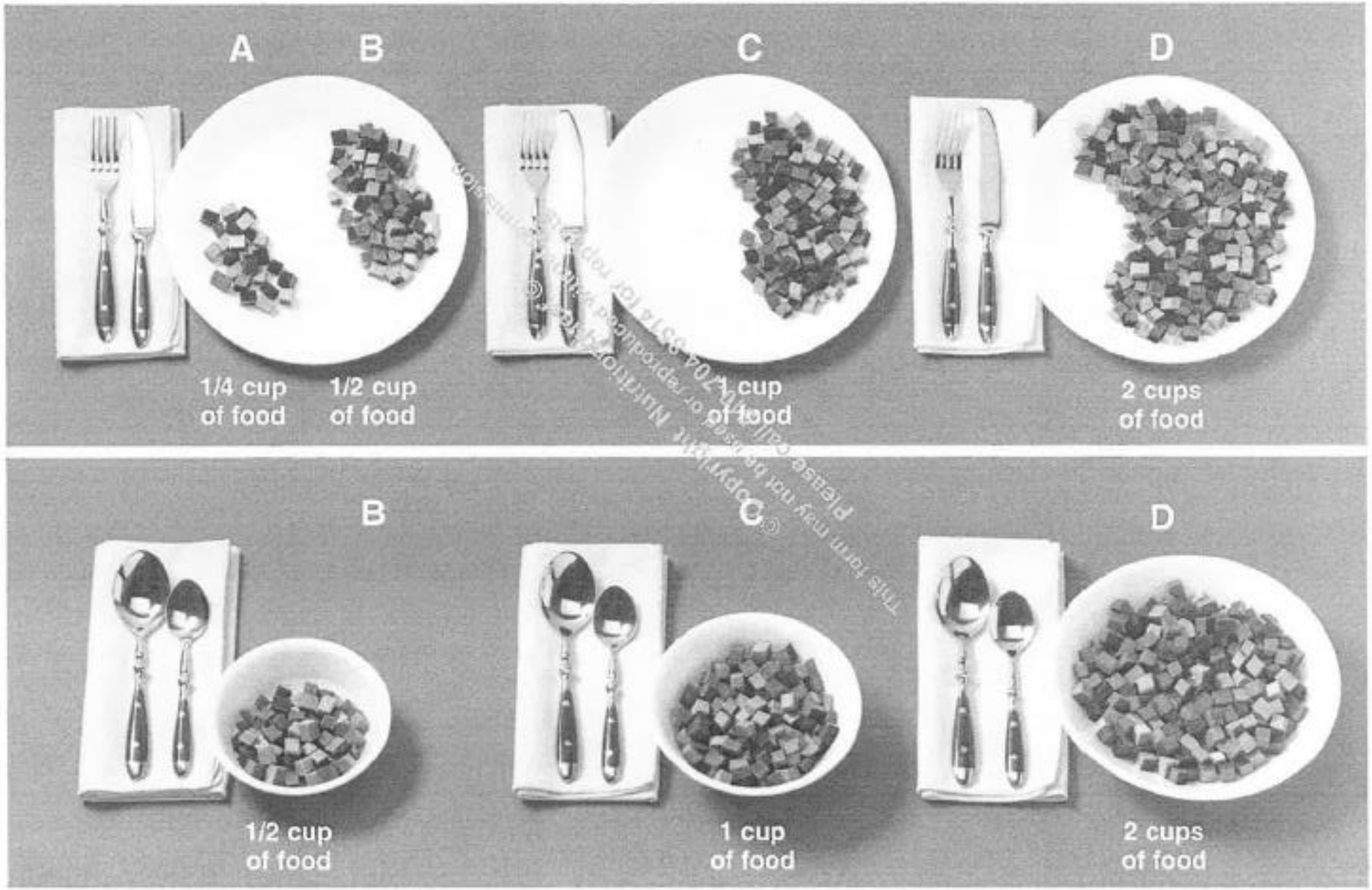

© Copyright Nutrition ... This form may not be used or reproduced without permission. Please call ... 704 ... 8514 for reprints.
